# A comprehensive protocol for quantitative magnetic resonance imaging of the brain at 3 Tesla

**DOI:** 10.1371/journal.pone.0297244

**Published:** 2024-05-31

**Authors:** Dvir Radunsky, Chen Solomon, Neta Stern, Tamar Blumenfeld-Katzir, Shir Filo, Aviv Mezer, Anita Karsa, Karin Shmueli, Lucas Soustelle, Guillaume Duhamel, Olivier M. Girard, Gal Kepler, Shai Shrot, Chen Hoffmann, Noam Ben-Eliezer

**Affiliations:** 1 Department of Biomedical Engineering, Tel-Aviv University, Tel Aviv, Israel; 2 The Edmond and Lily Safra Center for Brain Sciences, The Hebrew University of Jerusalem, Jerusalem, Israel; 3 Department of Medical Physics and Biomedical Engineering, University College London, London, United Kingdom; 4 Aix Marseille University, CNRS, CRMBM, Marseille, France; 5 Sagol School of Neuroscience, Tel Aviv University, Tel Aviv, Israel; 6 School of Neurobiology, Biochemistry and Biophysics, Faculty of Life Science, Tel Aviv University, Tel Aviv, Israel; 7 Sackler School of Medicine, Tel-Aviv University, Tel-Aviv, Israel; 8 Department of Diagnostic Imaging, Sheba Medical Center, Ramat-Gan, Israel; 9 Center for Advanced Imaging Innovation and Research (CAI2R), New-York University Langone Medical Center, New York, NY, United States of America; Pisa University Hospital, ITALY

## Abstract

Quantitative MRI (qMRI) has been shown to be clinically useful for numerous applications in the brain and body. The development of rapid, accurate, and reproducible qMRI techniques offers access to new multiparametric data, which can provide a comprehensive view of tissue pathology. This work introduces a multiparametric qMRI protocol along with full postprocessing pipelines, optimized for brain imaging at 3 Tesla and using state-of-the-art qMRI tools. The total scan time is under 50 minutes and includes eight pulse-sequences, which produce range of quantitative maps including T_1_, T_2_, and T_2_* relaxation times, magnetic susceptibility, water and macromolecular tissue fractions, mean diffusivity and fractional anisotropy, magnetization transfer ratio (MTR), and inhomogeneous MTR. Practical tips and limitations of using the protocol are also provided and discussed. Application of the protocol is presented on a cohort of 28 healthy volunteers and 12 brain regions-of-interest (ROIs). Quantitative values agreed with previously reported values. Statistical analysis revealed low variability of qMRI parameters across subjects, which, compared to intra-ROI variability, was x4.1 ± 0.9 times higher on average. Significant and positive linear relationship was found between right and left hemispheres’ values for all parameters and ROIs with Pearson correlation coefficients of *r*>0.89 (*P*<0.001), and mean slope of 0.95 ± 0.04. Finally, scan-rescan stability demonstrated high reproducibility of the measured parameters across ROIs and volunteers, with close-to-zero mean difference and without correlation between the mean and difference values (across map types, mean *P* value was 0.48 ± 0.27). The entire quantitative data and postprocessing scripts described in the manuscript are publicly available under dedicated GitHub and Figshare repositories. The quantitative maps produced by the presented protocol can promote longitudinal and multi-center studies, and improve the biological interpretability of qMRI by integrating multiple metrics that can reveal information, which is not apparent when examined using only a single contrast mechanism.

## 1 Introduction

Magnetic resonance imaging (**MRI**) technology constitutes one of the most efficient and versatile tools for investigating anatomical structures and physiological processes in vivo. Traditionally, contrast-weighted MR images are interpreted visually, while the absolute images’ intensity is arbitrary. Quantitative MRI (**qMRI**) provide an objective and efficient way to represent data, where numeric values of the tissue properties are being measured [1]. The ensuing quantitative markers can be used to probe both macro- and microscopic information while optimally utilizing the dynamic range of MRI contrast mechanisms [2, 3]. qMRI offers three main advantages. Versatility: the ability to generate parametric maps alongside traditional contrast-weighted images [4]; sensitivity: improved discernibility of tissue changes, e.g., alternation in relaxation time might be visually invisible yet quantitatively measurable [5]; and scalability, in the sense that qMRI extracts reproducible values, thereby facilitating longitudinal and multi-center studies [1, 6].

To date, qMRI is being used to measure a wide range of MR properties. These include: **T**_**1**_, **T**_**2**_, and **T**_2_^*^ relaxation times; proton-density (**PD**); magnetization transfer ratio (**MTR**) [7], inhomogeneous MTR (**ihMTR**) [8], MT saturation (**MTsat**) [9]; quantitative susceptibility maps (**QSM**) [10]; mean diffusivity (**MD**); fractional anisotropy (**FA**) [11]; water fraction (**WF**); and macromolecular tissue volume fraction (**MTVF**) [12]. These parameters reflect the tissue’s microarchitecture and biochemical state [13] and provide valuable information regarding its integrity and pathological state.

The clinical utility of qMRI has been demonstrated on numerous pathologies and body organs. In the context of brain research, qMRI has been used to study natural brain maturation [13–17] and to differentiate these from pathologies like multiple sclerosis (**MS**) [5, 18] and Alzheimer’s disease (**AD**) [19]. qMRI also exhibits superiority in tracking demyelinating processes in MS, mild cognitive impairment, and dementia [20, 21]; progressive atrophy and loss of function of neurons in AD, Parkinson’s disease, Wilson’s disease, Huntington’s disease, spinocerebellar ataxia, and myotonic dystrophy [22]; tumor development and infiltration [23, 24]; and to observe neurological changes in the presence of obesity [25] and diabetes [26]. Another example for the utility of qMRI it its use for improving the characterization of complex brain regions such as the Basal Ganglia and Midbrain [27].

The auspicious capabilities of qMRI comes with a tradeoff of longer scan-times needed to collect quantitative data, and the need for advanced signal models and processing tools in order to produce standardized cross-platform values. Choosing the optimal qMRI protocol for a specific application, however, is not trivial, considering the large variety of pulse sequences and postprocessing methods available [1, 28], and the need to account for hardware related bias like inhomogeneity of the main magnetic field B_0_, or the transmit (B_1_^+^) and receive (B_1_^–^) fields.

qMRI tools are being constantly improved, aiming to enhance their accuracy and reproducibility by optimizing acquisition strategies and data processing schemes [12, 29–32]. Some of these tools are designed to acquire multiparametric data, thereby covering multiple aspects of the tissue in a single scan session. Carter et al. [33], for example, used T_1_, T_2_*, MTsat, PD, FA, and MD maps to show that combinations of MR parameters may uncover microstructural information that is hidden when using individual metrics. Another study by Filo et al. [17] presented a multidimensional approach for characterization of aging-related changes associated with alterations in the molecular composition of the brain based on T_1_, T_2_, MTsat and MTVF maps. Schneider et al. [27] developed a machine learning algorithm, trained to classify the multiparametric data (QSM, MTR, and T_1_ maps) to characterize 21 subcortical nuclei structures. Tabelow et al. [34] developed a toolbox for multiparametric qMRI, termed *hMRI*, aiming to improve the accessibility and standardization in the field. This toolbox produces T_1_, T_2_*, PD and MTsat maps, calculates the white matter g-ratio [35], and integrates with other tools such as SPM [36] for estimating diffusion parameters. Lastly, some studies focus on improving standardization by investigating the reproducibility and repeatability of multiparametric protocols across MRI vendors and research centers [6, 32, 37].

In this study we present a comprehensive qMRI brain protocol, consisting of carefully chosen state-of-the-art pulse sequences and postprocessing pipelines. The protocol is used to produce ten quantitative maps: T_1_, T_2_, T_2_*, QSM, WF, MTVF, MD, FA, MTR, and ihMTR, with a total scan time of under 50 minutes for full brain coverage. Most importantly, we provide practical tips and limitations for each sequence and processing technique. Application of the ensuing multiparametric qMRI protocol is presented for a cohort of 28 healthy volunteers, for which quantitative values were estimated across 12 brain regions-of-interest (ROIs), and across two scan sessions in order to estimate their reproducibility. Additional statistical analysis included evaluation of inter-subject variability, intra-ROI variability, and correlation between right and left hemispheres.

## 2 Methods

### 2.1 Study population

Twenty-eight healthy volunteers ages 30–50 y/o (39.4 ± 5.7 y/o, 12 females) were scanned after obtaining informed written and verbal consent. The study was approved by the Helsinki committee of Sheba Medical Centre (3933-17-SMC). The written approval was signed by all volunteers prior to joining the study, confirming they meet the study inclusion criteria. These included daily drug consumption, chronic disorders (physical or mental), use of psychoactive substances, face, neck, or head tattoos (including permanent makeup), and deafness. Anatomical scans of all volunteers were examined by an expect neuroradiologist with >10 years of experience to rule out any incidental radiologic findings which did not manifest clinically. One volunteer (female, 38 y/o) was excluded from the study due to incidental finding in the white-matter (**WM**). Scan-rescan validations included twenty-three volunteers (39.7 ± 5.8 y/o, 10 females), which returned for a second, identical, scan session 30 ± 13 days after the first scan.

### 2.2 MRI scans and generation of quantitative maps

All Brain scans were performed on a 3T Siemens MAGNETOM Prisma scanner, using a 64-channels head and neck coil. Scans included eight pulse-sequences, that were used to produce ten types of quantitative maps. Nominal scan time was 45:30 min with an overhead of approximately 5–10 minutes for localizer scans and other calibrations. The full list of pulse sequences and scan settings is detailed in Table 1. We note that in the Discussion Section of this paper, we provide justifications for the selection of certain parameters provided under Table 1.

**Table 1:** List of pulse-sequences and scan parameters.

[#] Sequence	TR	TE	TI	Flip Angle (Inv. / Ref.)	Pixel BW	Voxel Resolution	Slab coverage	Accel. factor PE	Comments	Duration
↓	[ms]	[ms]	[ms]	[°]	[Hz/Px]	[mm^3^]	[mm]	(Ref. lines)		[mm:ss]
**[#1]** 2D Turbo-IR (FLAIR)	8000	81	2370	150	260	0.7x0.7x4.0	208	2 (24)	Concatinations = 2; Strong fat saturation; Turbo factor = 16	01:54
**[#2]** 3D Turbo-flash (MP2RAGE)	4000	3.52	716, 2180	5	220	1.0x1.0x1.0	192	2 (26)			06:00
**[#3]** 2D Multi-echo Spin-Echo (MESE)	5000	12:12:144	-	160	200	1.0x1.0x3.0	96	2 (24)	Gradient mode = fast	07:35
**[#4]** 3D Multi-echo gradient-echo (GRE)	41	5.2:4.5:36.9	-	15	310	1.0x1.0x1.0	128	2 (24)	Readout mode = Monopolar; Slice partial Fourier = 6/8	05:50
**[#5]** 2D IR SE echo planner imaging (EPI)	3270	49	200, 400, 1200, 2400	180	1776	2.0x2.0x3.0	132	2 (62)	Concatinations = 5; Weak fat saturation; EPI factor = 128	04:20
**[#6]** 3D Spoiled-GRE	26	3.23	-	5, 12, 27	430	1.0x1.0x2.2	132	2 (24)	Slice partial Fourier = 6/8	06:36
**[#7]** 2D Accelerated EPI sequence (CMRR)	3500	62.8	-	160	1886	2.0x2.0x2.0	144	-	b values = 0, 1000 s/mm^2^; Weak fat saturation; Multi-band accel. Factor = 2; 64 directions; phase-encoded (PE) direction = A>>P	03:58
									Diffusion data in 6 directions were collected with reversed PE direction = P>>A	00:35
**[#8]** 3D GRE + preparation pulses (ihMT^8^)	345	2.62	-	4	240	2.0x2.0x2.0	128	2 (32)	8 Tukey ihMT pulses: offset freq. = 7kHz, duration = 1ms, Flip angle = 260°, cosine fraction = 0.3 repeated every 1.5ms. 50% partial Fourier saturation was also used [8].	08:42
										**45:30**

Table 2 lists the types of contrast-weighted images produced by the sequences in Table 1, alongside the postprocessing techniques and ensuing qMRI maps. All data processing was performed on a 36-core computation server, running a Ubuntu 18.04 Linux operating system. Processing software included MATLAB R2020b (The MathWorks Inc., Natick, MA), a GNU C++ Compiler Collection (GCC) version 7.5, and Python versions 2.7 and 3.9. All processing pipelines are available online and can be downloaded from the URLs provided in the Data and code availability statements Section (request for download/use require either academic license, or a license for non-academic or commercial use).

**Table 2:** qMRI postprocessing techniques and resulting qMRI maps. Processing pipelines are available online and can be downloaded from the URLs provided in the Data and code availability statements Section.

Sequence	Output images	Post-processing method	Third party related softwares / code packages	Quantitative output maps
**[#3]** 2D Multi-echo Spin-Echo (MESE)	T_2_-weighted (see Fig 2C)	Echo Modulation Curve (EMC) [41, 42]	MP-PCA denoising [44, 45]	T_2_ (see Fig 3B}
**[#4]** 3D Multi-echo gradient-echo (GRE)	T_2_*-weighted (magnitude + phase images, see Fig 2D and 2E)	**T**_2_*: mono-exponential fitting **QSM**: Iterative Tikhonov regularization [47]	**1. FSL:** Masking and Rotating [48, 51–53] **2. MEDI toolbox:** nonlinear field fitting, phase unwrapping, Background field removal [53]	T_2_*, QSM (see Fig 3C, 3F)
**[#5]** 2D IR SE echo planner imaging (EPI)	T_1_-weighted (multi TI) (see Fig 2F)	mrQ software [12]	**1. FSL:** brain masks [48] **2. SPM8 + ANTs:** linear and non-linear image registrations [36, 58]	T_1_, WF, MTVF (see Fig 3A, 3D and 3E)
**[#6]** 3D Spoiled-GRE	T_1_-weighted (multi Flip Angle) (see Fig 2G)			
**[#7]** 2D Accelerated EPI sequence (CMRR)	Diffusion-weighted (collected with reversed phase-encoded directions) (see Fig 2H and 2I)	DWIPrep pipeline [64]	**1. ANTs**: correction of intensity non-uniformity and brain masking [66, 67] **2. FSL:** Boundary-Based registration, and susceptibility distortion, Eddy current and motion corrections [52, 70, 71] **3. MRtrix3**: MP-PCA denoising, B_1_^+^ inhomogeneity correction, diffusion tensor images calculation (dwi2tensor and tensor2metric) [68, 69, 72, 73]	MD, FA (see Fig 3G and 3H)
**[#8]** 3D GRE + preperation pulses (ihMT^8^)	MT weighted (multiple frequencies) (see Fig 2J–2N)	ihMT pipeline [77]	1. **FSL**: brain masks [48] 2. **MRtrix3:** MP-PCA denoising [68] 3. **ihMT-MoCo:** Gibbs and motion artifacts corrections [78]	MTR, ihMTR (see Fig 3I and 3J)

#### 2.2.1 Anatomical scans: FLAIR and MP2RAGE

Brain protocol included two *qualitative* anatomical scans: a fluid attenuation inversion recovery (**FLAIR** [38], Sequence #1), and an MP2RAGE scan (Sequence #2) which was used for image segmentation. The MP2RAGE protocol is based on a 3D magnetization-prepared rapid gradient-echo (**MPRAGE**) sequence, modified to generate images at two different inversion times (**TI**s). MP2RAGE images are free from B_1_^+^ inhomogeneities, T_2_ contrast, and overall offers superior delineation between WM and GM tissues, leading to improved segmentation capabilities [39, 40]. This pair of anatomical scans were required by the IRB committee, to be reviewed by an expert neuroradiologist in order to overrule accidental radiologic findings.

#### 2.2.2 Transverse relaxation time- T_2_

T_2_ maps were produced from multi-echo Spin-echo (**MESE**) data (Sequence #3) using the Echo Modulation Curve (**EMC**) algorithm [41, 42]. Accuracy of EMC-derived T_2_ values was validated using reference physical phantoms and in vivo, producing high accuracy and reproducibility across the physiological range of T_2_ values and across scan settings [43]. Additional pre-processing included Marchenko-Pastur Principal Component Analysis (**MP-PCA**) denoising [44, 45].

#### 2.2.3. Observed transverse relaxation time- T_2_*, and quantitative susceptibility mapping

Quantitative T_2_* maps were generated using a 3D multi-echo spoiled GRE (i.e., **FLASH**) protocol [46] (Sequence #4) assuming a mono-exponential decay pattern. T_2_* values were calculated as the slope of a linear regression of the log of the signal magnitude. To avoid measurement bias due to Rician noise distribution, data points whose magnitude was below 10% of the 1^st^ echo intensity were truncated from the signal time-series and excluded from the fit.

Magnitude and phase images were used to calculate quantitative susceptibility maps (**QSM**), based on the pipeline described by Karsa et al. [47]. The reconstruction process included conversion of images from DICOM to NIFTI format, brain masking using FSL’s Brain Extraction Tool (**BET**) [48], and noise-based mask erosion [49]. Nonlinear fitting of the complex data [30] was used to obtain a total field map, followed by Laplacian phase unwrapping [50]. The total field map, brain mask, and noise map obtained from the nonlinear fitting process, were aligned to the B_0_ direction using FSL’s FLIRT tool [51–53]. Imaged FOV was increased prior to rotation by using zero padding in the image domain to ensure that images are not cropped during this process. Projection onto Dipole Fields (**PDF**) [54] was used to remove background fields. The nonlinear field fitting, Laplacian phase unwrapping, and background field removal were based on functions from Cornell’s MEDI toolbox [55]. Lastly, susceptibility maps were calculated using iterative fitting in the image domain with Tikhonov regularization (regularization weight α = 0.05) [47]. A pipeline with the full set of postprocessing steps is available at: https://xip.uclb.com/product/mri_qsm_tkd.

#### 2.2.4 Longitudinal relaxation (T_1_), water fraction, and macromolecular tissue volume fraction

T_1_ relaxation and related parametric maps were processed from the variable flip angle single-echo FLASH sequence data (Sequence #6) using the mrQ software package [12]. T_1_ and PD values produced by the mrQ pipeline showed high accuracy and reliability compared to reference values for both phantoms and in vivo models [12, 56]. To correct for B_1_^+^ inhomogeneity, low-resolution inversion recovery echo-planner imaging (**IR-EPI**) data was provided as an input to the fitting process (Sequence #5).

Following conversion of the DICOM images to NIFTI format, the mrQ software reconstructs two T_1_ maps: the first from the series of multi flip-angle FLASH images, and the second from IR-EPI data, according to Barral et al. [57]. Processing steps included brain masking (FSL’s BET [48]), linear registration between the IR-EPI images using SPM8 [36], and non-linear registrations of the FLASH and IR-EPI data using ANTs [58]. We note that the mrQ processing pipeline also uses open-source code repositories such as Vistasoft [59], KNKUtils [60] and nimsdata [61].

During postprocessing, B_1_^+^ profile was calculated as follows. First, the low resolution IR-EPI data were used to calibrate the nominal RF excitation flip angles [12]. T_1_ values were calculated from this data and were provided as input for the multi-flip-angle FLASH signal model [57], allowing to extract the true flip angle, which deviates from the nominal values due to inhomogeneity of the B_1_^+^ field. Actual B_1_^+^ values were then calculated as the ratio between the true and the nominal flip angles and were used to correct the T_1_ values of the high resolution multi flip-angle FLASH data.

In addition to T_1_ maps, the mrQ pipeline produced a water fraction (**WF**) map, calculated as the ratio between white or gray matter and the CSF’s proton densities, and the macromolecular tissue volume fraction (**MTVF**) map, which is equal to the remaining tissue fraction (i.e., MTVF ≜ 1-WF) [12].

#### 2.2.5 Diffusion tensor maps: Mean diffusivity and Fractional anisotropy

Diffusion weighted images (**DWI**) were collected using a 2D multi-slice multi-band accelerated EPI sequence (Sequence #7), developed at the Centre for Magnetic Resonance Research (CMRR, University of Minnesota). Two scans were performed, one with phase-encoding (**PE**) along the posterior-to-anterior (P>>A) direction, and the second with reversed (A>>P) direction, for later susceptibility distortion correction (**SDC**) [62].

Anatomical and DWI data was organized in brain imaging data structure (**BIDS**) format [63], and processed according to the DWIPrep pipeline [64], which includes co-registration between the low-resolution DWI data and the high-resolution MP2RAGE anatomical data. MP2RAGE T_1_-weighted (**T**_**1**_**w**) anatomical data preprocessing was performed using sMRIPprep [65], where images are corrected for intensity non-uniformity (**INU**) using ANTs N4ITK [66, 67], and skull-stripped using ANTs ‘antsBrainExtraction’ workflow.

DWI data preprocessing included MP-PCA denoising using MRtrix3’s dwidenoise [68, 69]. FSL’s *topup* [52] and *eddy* [70] functions were applied for motion and susceptibility distortion corrections using the pair of A>>P and P>>A phase encoded images (b = 0). Lastly, nonuniformity of the receiver gain was corrected using MRtrix3’s N4ITK [66, 68]. A preprocessed reference b = 0 image was used for the co-registration process between diffusion and anatomical data. This co-registration was performed using Boundary-Based registration [71] as implemented in FSL’s *epi_reg*. The resulting transformation matrix was applied to all preprocessed diffusion images, producing images that match the subjects’ MP2RAGE space.

Diffusion tensor images (**DTI**) were calculated from the preprocessed diffusion images using MRtrix3’s dwi2tensor. Finally, the DTI-derived maps, MD, and FA, were extracted using MRtrix3’s tensor2metric [72, 73]. We note that due to the relatively small matrix size of the original DWI data (106x106), quantitative DTI values were calculated in the MP2RAGE space, i.e., the diffusion data was interpolated and then registered to T_1_-weighted MP2RAGE space, a common procedure for studies in the field [74–76].

#### 2.2.6 Magnetization transfer (MT) maps: MTR and ihMTR

MT weighted data were collected using an ihMT sequence, consisting of bursts of MT preparation pulses interleaved with multiple GRE readouts acquired using a 3D pulse-sequence (Sequence #8) as introduced in Mchinda et al. [8]. This sequence relies on frequency alternation, producing the desired contrast of dual-saturation which is required to generate ihMT data. The protocol was further optimized for 3T brain applications by Soustelle et al. [77] so that the derived ihMTR maps are immune to B_1_^+^ heterogeneity. Other scan settings included non-selective RF excitation, coronal slice orientation, and three averages to improve SNR. The final dataset consisted of five types of images: (1) unsaturated volume, *S*_0_; (2) single-offset positive +Δ*f*, *S*^+^; (3) dual-offset alternated ±Δ*f* saturation, *S*^±^; (4) single-offset negative −Δ*f* saturation, *S*^−^; and (5) opposite dual-offset alternated ∓Δ*f* saturation, *S*^∓^.

The ihMT processing pipeline is described in Soustelle et al. [78]. Preprocessing included masking using FSL’s BET [48] tool, MP-PCA denoising using MRtrix3 [68], Gibbs artifacts correction with cosine apodization, and motion compensation using ihMT-MoCo [78]. A pipeline with the prescribed postprocessing steps is available at: https://github.com/lsoustelle/ihmt_proc. Processed images were then used to calculate the MT-related quantitative maps according to Duhamel et al. [79]. In this work, we present two MT-related maps: the MTR ≜ 1−*S*^+^/*S*_0_, and ihMTR ≜ [(*S*^+^+*S*^−^)−(*S*^±^+*S*^∓^)]/*S*_0_, which is known for its high sensitivity to myelin content [18, 79, 80].

### 2.3 Segmentation of brain regions of interest

Images were segmented using FreeSurfer’s *recon-all* function [81], which performs full brain reconstruction from anatomical T_1_w MP2RAGE data. Segmentations were then used for extracting values from the T_2_, T_2_*, QSM, MTR, ihMTR, MD, and FA maps. Linear registration of the MP2RAGE segments onto the T_2_, T_2_*, QSM, MTR and ihMTR maps-space was performed using FreeSurfer tools [71]. For the Diffusion maps, MD and FA, registration was performed in the opposite direction, i.e., the diffusion images were projected into the MP2RAGE space, as described under the DWI processing Section above.

Separate segmentation was performed for the mrQ maps (T_1_, WF, and MTVF). This separation simplifies the segmentation of mrQ output maps, whose processing pipeline involves non-linear registration between FLASH and EPI data in a coordinate space which is different than the MP2RAGE. Thus, in order to segment these maps, synthetic T_1_w images were generated from mrQ T_1_ and PD maps based on the Ernst equation [9], and then used as input to FreeSurfer’s recon-all function.

FreeSurfer was previously shown to have high scan-rescan stability across subjects, scan sessions and pulse-sequences schemes [82, 83]. Nevertheless, to assess differences in ROIs segmentation between the two segmented datasets used in the current study (i.e., synthetic T_1_w and MP2RAGE), we compared the volume of each ROI (i.e., number of voxels in ROI x voxel volume), and calculated the mean ± SD percent of change in each ROI volume across volunteers.

**Fig 1:**
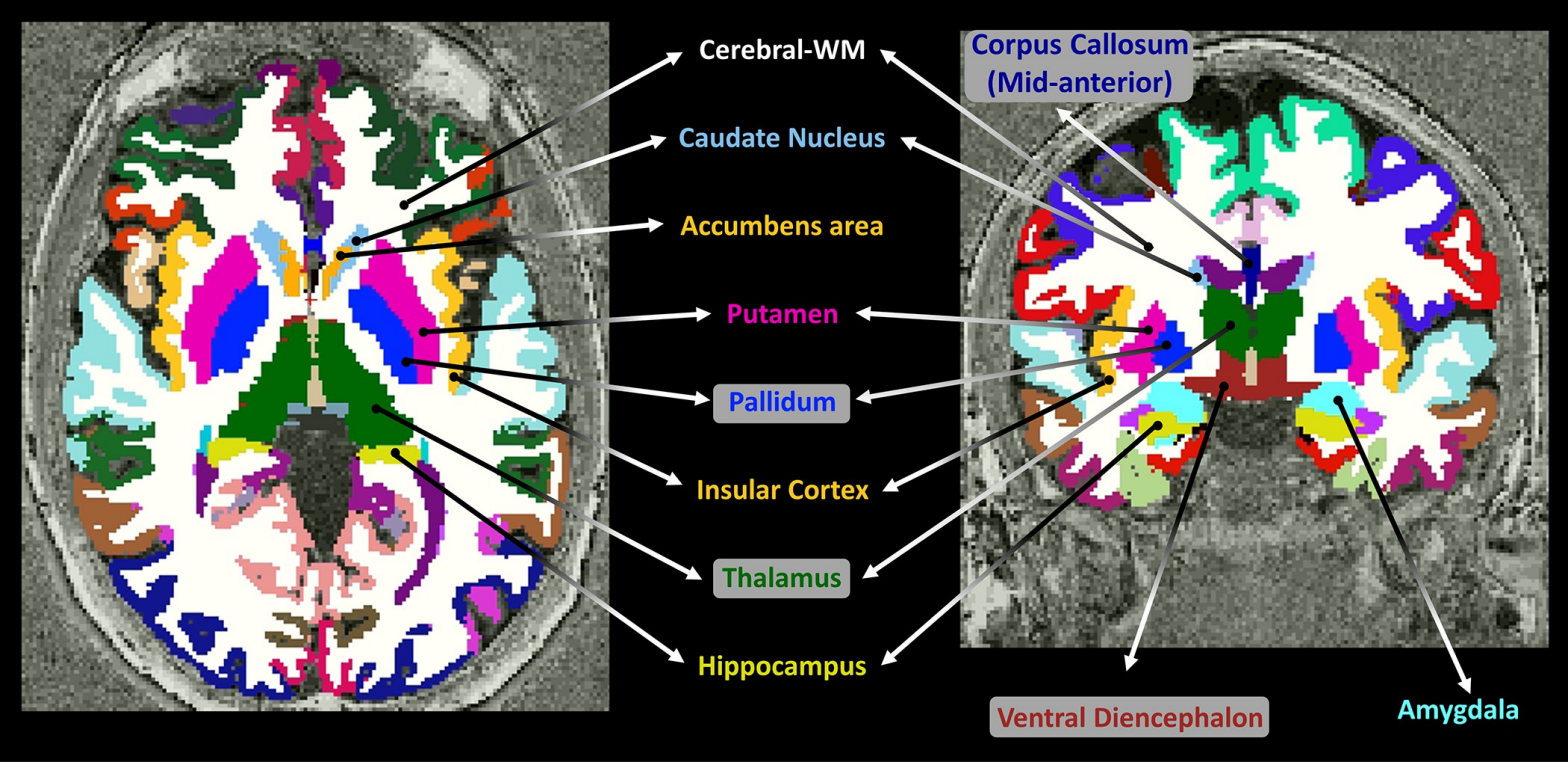
FreeSurfer segmented ROIs, overlaid on axial (left) and coronal (right) T_1_-weighted MP2RAGE images.

Segmented ROIs were used to extract mean ± SD values for each segment and for each map. To reduce partial volume effects, a single-voxel erosion was applied on the segmentation map to each ROI, followed by a weak Chauvenet’s criterion [84] to exclude outlier voxels from the qMRI map, using a 3 x SD threshold. ROIs included the cerebral-WM, caudate nucleus (**CN**), putamen, pallidum (i.e., the globus pallidus), corpus callosum (**CC**), thalamus, ventral diencephalon (ventral **DC**), accumbens area (i.e., nucleus accumbens), amygdala, hippocampus, insular cortex, and cortex-all (i.e., all cortical structures). An example for Freesurfer segmentation of MP2RAGE data, including the investigated brain regions, is given in Fig 1.

### 2.4 Statistical analysis

Statistical analysis included estimation of inter-subject and intra-ROI variability, correlation between right and left hemispheres, and scan-rescan reproducibility. Mean ± SD of the number of voxels and qMRI values were calculated for each ROI and for each quantitative map. Importantly, the qMRI maps, rather than segmentation maps, were used for calculating the number of voxels within each ROI. This means that in voxels with a value of zero in the qMRI maps (e.g., due to fitting errors, masking, or other processing step like background removal for QSM maps) were not included in the statistical analysis. SD of the mean values across subjects represents the inter-subject variability, while the mean of SD represents the average intra-ROI variability. Coefficient of variation (CV) was calculated as the ratio between the SD and mean (i.e., CV = 100% * SD/mean), reflecting the variability of the relevant measurand. Additional analysis was performed to evaluate the percentage of outlier voxels in each quantitative map. This was done by calculating the number of voxels and mean qMRI values across volunteers also without removal of outlier voxels. The relative change in number of voxels across volunteers was then calculated between the two datasets (i.e., without and with voxels removal using weak Chauvenet’s criterion with 3xSD threshold).

Correlation between quantitative values in the right and left hemispheres was estimated using linear regression, producing equation of linearity, Pearson’s correlation coefficient *r*, and the *P*-value. The corpus callosum was excluded from the right-left analysis since FreeSurfer does not differentiate between its right and left segments.

Scan-rescan analysis was done on data from 23 volunteers who came back for a repeated scan 30±13 days after the first scan. Bland-Altman plots were generated for each map and across all investigated ROIs, comparing the mean values from the two scan sessions. Lastly, the Bland-Altman plots were used to calculate the mean ± SD of the difference between the two sessions, limits of agreement, and the linear correlation between the sessions’ difference and mean.

## 3 Results

### 3.1 Contrast-weighted images and quantitative maps

**Fig 2:**
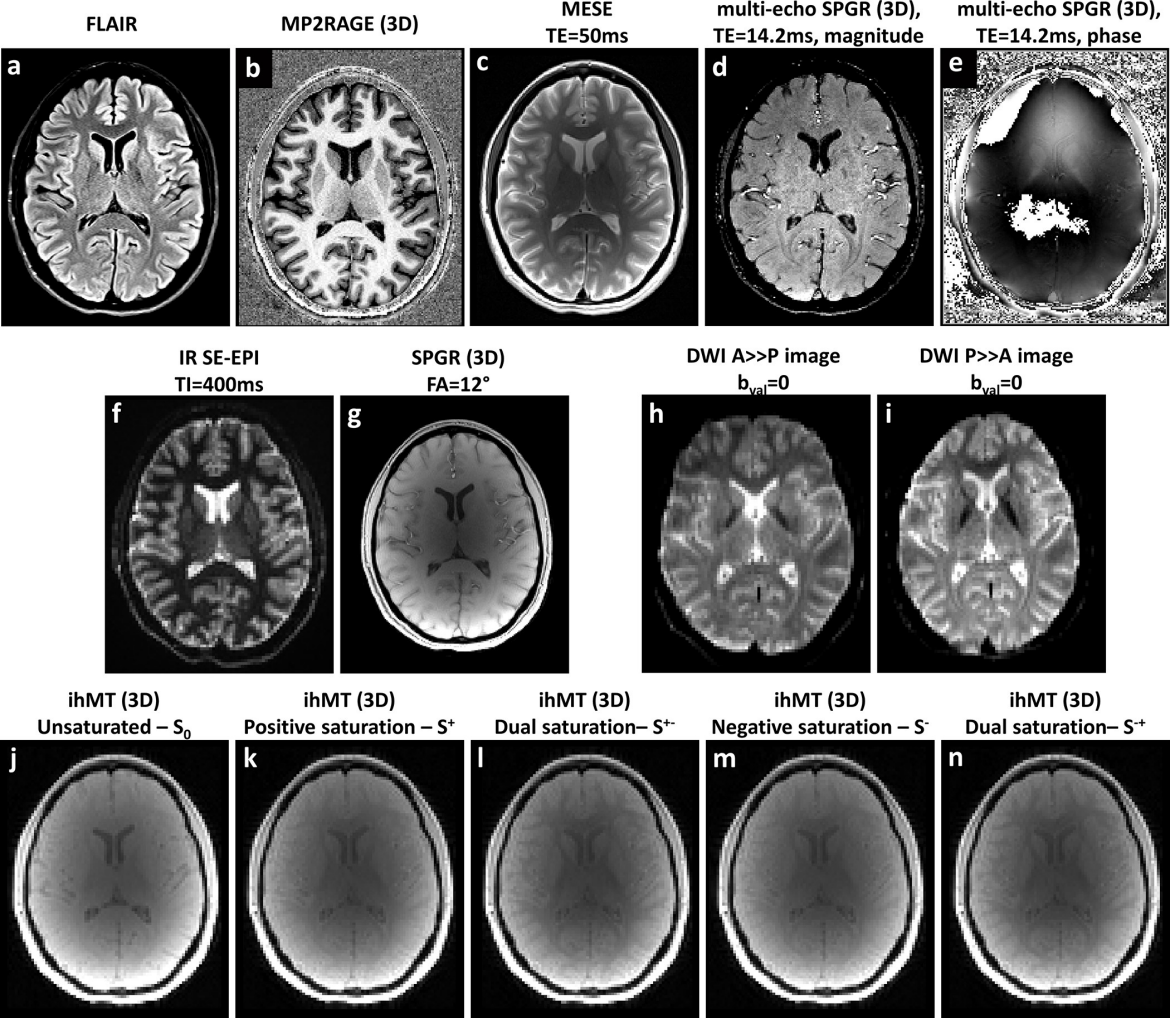
Sample contrast-weighted images for a single volunteer (F, 31 y/o). **(a, b)** Anatomical scans using FLAIR and MP2RAGE scans. Relaxation weighted images include (**c**) T_2_-weighted multi-echo SE (MESE); (**d, e**) T_2_* weighted multi-echo Spoiled-GRE (TE = 14.2 ms) magnitude and phase images; (**f**) T_1_-weighted inversion recovery SE-EPI; and (**g**) T_1_-weighted single-echo spoiled-GRE (flip angle = 12°). (**h, i**) Diffusion-weighted images acquired using two phase-encoding directions: anterior-to-posterior and posterior-to-anterior. Inhomogeneous MT (ihMT) images: (**j**) Unsaturated M_0_, (**k**) single positive saturation S^+^, (**l**) Dual saturation S^+-^, (**m**) single negative saturation S^-^, and (**n**) Dual saturation S^-+^.

Representative contrast-weighted images from all sequences are shown in Fig 2 for a single volunteer (F, 31 y/o). The figure shows 2D axially reformatted images at the level of the lateral ventricles. Anatomical images include FLAIR (Fig 2A, Sequence #1) and MP2RAGE (Fig 2B, Sequence #2). Fig 2C (MESE, Sequence #3) is shown for TE of 50 ms and Fig 2D and 2E (multi-echo 3D FLASH, Sequence #4) are shown for TE of 14.2 ms; images from other echo times are not shown. T_1_-weighted images are shown in Fig 2F and 2G. The IR SE-EPI image (Fig 2F, Sequence #5) was acquired using TI = 400 ms, and the FLASH image (Fig 2G, Sequence #6) using flip angle = 12° (other TIs and flip angles are not shown). Diffusion weighted images (Sequence #7) are shown in Fig 2H and 2I for b-value = 0 and both PE directions. Lastly, raw ihMT images (Sequence #8) are shown in Fig 2J–2N and include the unsaturated image *S*_0_, and the four types of saturated images, denoted as *S*^+^, *S*^−^, *S*^±^ and *S*^∓^. For completeness, DWI data with b-value≠0 is given as a supplementary S1 Fig, corresponding to Fig 2H and 2I.

**Fig 3:**
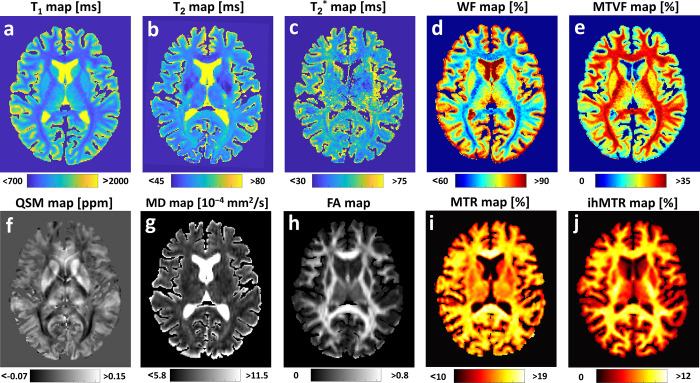
Example of quantitative maps from a single volunteer (F, 31 y/o). Data were reformatted to show a 2D axial slice at the level of the lateral ventricles (i.e., similar slice across maps). Relaxation maps include (**a**) T_1_, (**b**) T_2_ and (**c**) T_2_*. Fractional tissue volumes: (**d**) water (WF) and (**e**) macromolecules (MTVF). (**f**) Quantitative susceptibility map (QSM). Diffusion tensor images: (**g**) Mean diffusivity (MD) and (**h**) Fractional anisotropy (FA). Magnetization transfer (MT) related maps: (**i**) MT ratio (MTR) and (**j**) inhomogeneous MT ratio (ihMTR). Brain masks are based on FreeSurfer segmentations.

Representative quantitative maps are shown in Fig 3 for the same volunteer (F, 31 y/o), reconstructed from the raw data shown in Fig 2, and including the following quantitative maps [units]: T_1_ [ms], T_2_ [ms], T_2_* [ms], WF [%], MTVF [%], QSM [ppm], MD [10^−4^ mm^2^/s], FA [0…1], MTR [%], and ihMTR [%]. Brain masks were based on FreeSurfer segmentation. For visualization purposes, color maps were chosen to represent the different metrics: ‘Parula’–maps of relaxation times, ‘Jet’–tissue fraction maps, ‘Gray’ for QSM and diffusion maps, and ‘Hot’–MT-related maps.

We note that in the T_2_* maps (Fig 3c), regions with long relaxation times such as the blood vessels (e.g., choroidal vessels) and areas with CSF (e.g., lateral ventricle) appear as null. It was not possible to accurately quantify the long T_2_* values in these regions, since the maximal TE employed for fitting was too short (36.9 ms) [85].

### 3.2 Mapping of qMRI values in different brain ROIs

Table 3 delineates parametric values extracted for each ROI and quantitative map, averaged across all volunteers (N = 28). A 3D volume with both right and left hemispheres values were included in this analysis. The first three columns contain the mean ± SD of the number of voxels in each ROI, and the CV which reflects the variability of each ROI volume across subjects (e.g., the number of voxels in the Cerebral-WM region of the T_1_ map was 182915 ± 23808 reflecting a variability of 13.0% across subjects). Fourth to sixth columns contain the mean ± SD of measured qMRI value across volunteers, with SD representing inter-subject variability, and the CV represent variability of mean qMRI value across subjects. Intra-ROI variability (SD) of quantitative values is given in the seventh column, averaged across all volunteers. The mean ± SD number of voxels can be translated into volume in cubic millimeters of each ROI, by multiplying these values by the voxel volume of each quantitative map (see Table 1). For improved interpretation of the results, the qMRI values were also provided in box plots, see Supplementary S2 Fig. As a general comment, mrQ related results (i.e., T_1_, WF and MTVF maps) are highlighted in light grey color in all manuscript’s Tables to emphasize that two different datasets were used for segmentation (i.e., synthetic T_1_w for mrQ related results and MP2RAGE for all others).

**Table 3:** Quantitative values of ten MR parameters in twelve representative Brain regions: Cerebral-white-matter (WM), caudate nucleus (CN), putamen, pallidum (i.e., the globus pallidus), corpus callosum (CC), thalamus, ventral diencephalon (ventral DC), accumbens area (i.e., nucleus accumbens), amygdala, hippocampus, insular cortex, and cortex-all (i.e., all cortical structures). Values are presented for each qMRI map and brain ROI, across the 28 volunteers. The columns represent: mean ± SD of the number of voxels, mean ± SD of quantitative value (SD represents the inter-subject variability), and the average SD within the ROI (SD represents the intra-ROI variability). Coefficient of variation (CV) across volunteers was calculated by dividing the SD with the mean for both number of voxels and qMRI value. The mean ± SD number of voxels can be translated into volume in cubic millimeters of each ROI, by multiplying these values by the voxel volume of each quantitative map (see Table 1). Due to the different datasets used for segmentation, (i.e., synthetic T_1_w and MP2RAGE images), mrQ related results (namely, T_1_, WF and MTVF maps) are highlighted in grey.

Quantitative map type	ROI name	Mean number of voxels across volunteers			Mean qMRI values across volunteers			Intra-ROI variability
		Mean	SD	CV	Mean	SD	CV	
*T*_*1*_		[#]	[#]	[%]	[ms]	[ms]	[%]	[ms]
	Cerebral-WM	182915	23808	13	982	22	2	144
	Caudate Nucleous	1846	223	12	1335	43	3	111
	Putamen	2393	568	24	1273	61	5	109
	Pallidum	1008	291	29	1015	31	3	69
	Corpus callusom	1165	147	13	1051	42	4	254
	Thalamus	5499	1076	20	1195	35	3	151
	VentralDC	3430	707	21	1110	49	4	192
	Accumbens-area	296	74	25	1455	56	4	126
	Amygdala	1077	277	26	1405	137	10	195
	Hippocampus	2796	984	35	1411	120	9	212
	ctx_insula	3067	444	14	1524	79	5	203
	ctx	91671	11122	12	1423	40	3	291
*T*_*2*_		[#]	[#]	[%]	[ms]	[ms]	[%]	[ms]
	Cerebral-WM	121505	15084	12	58.8	1.4	2	4.8
	Caudate Nucleous	1802	195	11	57.9	2.0	3	7.7
	Putamen	2750	271	10	52.0	2.2	4	4.5
	Pallidum	963	135	14	42.0	1.5	4	6.3
	Corpus callusom	915	120	13	65.7	1.9	3	16.0
	Thalamus	4205	425	10	56.8	1.3	2	6.1
	VentralDC	2116	229	11	59.2	1.6	3	10.9
	Accumbens-area	261	38	14	64.6	1.7	3	6.0
	Amygdala	691	110	16	68.8	1.6	2	5.5
	Hippocampus	1855	208	11	71.7	2.3	3	8.5
	ctx_insula	2719	326	12	69.1	1.3	2	6.9
	ctx	69895	6537	9	66.4	1.7	3	8.7
*T*_2_*		[#]	[#]	[%]	[ms]	[ms]	[%]	[ms]
	Cerebral-WM	366941	46739	13	49.2	1.7	3	7.3
	Caudate Nucleous	5384	597	11	48.8	3.5	7	10.9
	Putamen	8215	811	10	44.2	3.8	9	9.8
	Pallidum	2909	403	14	29.5	2.4	8	7.6
	Corpus callusom	2710	365	13	50.5	2.8	5	11.8
	Thalamus	12433	1182	10	51.5	4.2	8	10.5
	VentralDC	6240	606	10	46.3	2.2	5	15.8
	Accumbens-area	764	98	13	59.1	6.4	11	14.6
	Amygdala	2027	255	13	67.4	5.0	7	17.4
	Hippocampus	5487	529	10	64.4	4.0	6	19.2
	ctx_insula	7756	926	12	67.9	2.7	4	16.9
	ctx	219014	17995	8	57.5	2.7	5	15.5
***QSM***		[#]	[#]	[%]	[10^−2^ ppm]	[10^−2^ ppm]	[%]	[10^−2^ ppm]
	Cerebral-WM	365817	48168	13	-0.76	0.39	-52	3.71
	Caudate Nucleous	4329	593	14	8.49	1.40	17	3.49
	Putamen	6982	826	12	7.65	1.97	26	5.21
	Pallidum	2579	416	16	20.14	2.46	12	7.22
	Corpus callusom	2026	255	13	3.40	1.18	35	4.76
	Thalamus	12370	1314	11	3.67	0.95	26	4.18
	VentralDC	6610	730	11	2.48	1.28	52	8.95
	Accumbens-area	684	109	16	1.53	2.83	185	5.01
	Amygdala	2509	272	11	-1.50	0.97	-65	3.68
	Hippocampus	6332	629	10	-0.55	1.17	-215	4.32
	ctx_insula	6878	903	13	0.35	0.69	195	3.93
	ctx	219908	19698	9	0.45	0.23	51	4.12
***WF***		[#]	[#]	[%]	[%]	[%]	[%]	[%]
	Cerebral-WM	182878	23798	13	70.5	0.8	1	4.5
	Caudate Nucleous	1842	222	12	79.6	1.0	1	2.3
	Putamen	2382	567	24	78.0	1.8	2	2.5
	Pallidum	1010	292	29	70.3	1.1	2	2.7
	Corpus callusom	1173	148	13	71.9	1.2	2	4.8
	Thalamus	5551	1084	20	75.7	0.9	1	3.7
	VentralDC	3461	716	21	73.1	1.3	2	5.1
	Accumbens-area	296	73	25	80.9	1.7	2	2.6
	Amygdala	1077	277	26	80.2	2.9	4	4.5
	Hippocampus	2801	984	35	80.0	2.9	4	4.6
	ctx_insula	3058	435	14	81.2	1.7	2	3.8
	ctx	91529	11105	12	79.5	1.1	1	10.1
***MTVF***		[#]	[#]	[%]	[%]	[%]	[%]	[%]
	Cerebral-WM	182875	23799	13	29.5	0.8	3	4.5
	Caudate Nucleous	1842	222	12	20.4	1.0	5	2.3
	Putamen	2382	567	24	22.0	1.8	8	2.5
	Pallidum	1010	292	29	29.7	1.1	4	2.7
	Corpus callusom	1173	148	13	28.1	1.2	4	4.8
	Thalamus	5551	1084	20	24.3	0.9	4	3.7
	VentralDC	3461	716	21	26.9	1.2	5	5.1
	Accumbens-area	296	73	25	19.1	1.7	9	2.6
	Amygdala	1077	277	26	19.8	2.9	15	4.5
	Hippocampus	2801	984	35	20.0	2.9	14	4.6
	ctx_insula	3058	435	14	18.8	1.7	9	3.8
	ctx	91500	11095	12	20.5	1.1	6	10.1
***MD***		[#]	[#]	[%]	[10^−4^ mm^2^/s]	[10^−4^ mm^2^/s]	[%]	[10^−4^ mm^2^/s]
	Cerebral-WM	363423	48393	13	7.37	0.15	2	0.52
	Caudate Nucleous	4261	579	14	8.14	0.43	5	1.99
	Putamen	6881	816	12	6.94	0.10	1	0.40
	Pallidum	2587	410	16	6.83	0.15	2	0.51
	Corpus callusom	2009	247	12	9.23	0.29	3	2.01
	Thalamus	12100	1295	11	7.59	0.16	2	1.17
	VentralDC	6467	719	11	8.02	0.48	6	2.09
	Accumbens-area	675	109	16	7.90	0.22	3	0.57
	Amygdala	2469	271	11	8.35	0.21	2	1.51
	Hippocampus	6240	619	10	8.78	0.32	4	1.45
	ctx_insula	6806	889	13	8.34	0.18	2	0.91
	ctx	223597	19826	9	8.55	0.22	3	1.52
***FA***		[#]	[#]	[%]	[unitless]	[unitless]	[%]	[unitless]
	Cerebral-WM	366939	48601	13	0.41	0.01	3	0.14
	Caudate Nucleous	4288	587	14	0.17	0.01	9	0.06
	Putamen	6940	816	12	0.20	0.01	8	0.08
	Pallidum	2609	415	16	0.33	0.03	8	0.12
	Corpus callusom	2041	255	12	0.61	0.03	5	0.17
	Thalamus	12327	1314	11	0.32	0.01	5	0.08
	VentralDC	6627	735	11	0.46	0.04	8	0.17
	Accumbens-area	678	107	16	0.23	0.04	16	0.07
	Amygdala	2496	271	11	0.19	0.01	7	0.06
	Hippocampus	6283	617	10	0.18	0.01	6	0.07
	ctx_insula	6861	901	13	0.18	0.01	7	0.06
	ctx	224493	20025	9	0.17	0.01	5	0.07
***MTR***		[#]	[#]	[%]	[%]	[%]	[%]	[%]
	Cerebral-WM	366874	49139	13	16.2	0.3	2	0.9
	Caudate Nucleous	4325	594	14	13.7	0.5	3	0.9
	Putamen	6947	822	12	14.1	0.4	3	0.7
	Pallidum	2608	415	16	14.6	0.4	3	0.7
	Corpus callusom	2023	250	12	16.3	0.4	2	1.7
	Thalamus	12200	1321	11	15.0	0.4	3	0.7
	VentralDC	6556	750	11	14.9	0.5	3	1.2
	Accumbens-area	682	108	16	13.0	0.3	3	0.6
	Amygdala	2495	277	11	14.7	0.4	3	0.9
	Hippocampus	6337	640	10	14.6	0.4	2	1.0
	ctx_insula	6876	905	13	14.4	0.3	2	1.1
	ctx	224340	20659	9	14.4	0.3	2	1.4
***ihMTR***		[#]	[#]	[%]	[%]	[%]	[%]	[%]
	Cerebral-WM	367002	48965	13	8.2	0.3	3	1.5
	Caudate Nucleous	4342	599	14	3.2	0.2	8	0.9
	Putamen	6957	822	12	4.0	0.3	6	0.9
	Pallidum	2601	417	16	6.5	0.5	7	1.2
	Corpus callusom	2034	254	12	7.9	0.4	5	1.6
	Thalamus	12408	1338	11	5.8	0.5	8	1.4
	VentralDC	6622	754	11	7.4	0.5	7	1.8
	Accumbens-area	684	110	16	3.0	0.3	11	0.7
	Amygdala	2510	276	11	4.1	0.3	7	1.0
	Hippocampus	6366	645	10	4.2	0.2	5	1.0
	ctx_insula	6907	925	13	3.4	0.2	5	0.9
	ctx	224059	20770	9	3.8	0.3	9	1.2

A notable finding that arises from Table 3 is that for most map types and ROIs, the intra-ROI variability (i.e., SD of values within each ROI) is higher than the inter-subject variability (i.e., SD of mean values across volunteers). On average, the intra-ROI variability was x(4.1 ± 0.9) times higher than the inter-subject variability.

We note that the values were also calculated separately for the right and left hemispheres and the full results are given in Table A in S1 Text under the supplementary materials. This Table does not include the Corpus callosum since the separation to hemispheres for this region is not supported by the FreeSurfer tool.

Two other types of analysis were performed to assess the percentage of excluded outlier voxels, and the segmentation consistency between the two FreeSurfer segmentation datasets (i.e., MP2RAGE and Synthetic-T_1_w spaces). For the first analysis, data were processed with and without removing outlier voxels, and the percentage of removed voxels and change in qMRI value were calculated per map type and ROI. This result is given in the supplementary Table B in S1 Text. The percentages of change in mean qMRI values were than averaged across ROIs (i.e., producing the average percentage of difference per map type across volunteers and ROIs). This analysis revealed a very small difference in the qMRI values, smaller than 1.5% for all map types besides the QSM results where a difference of 4.1% was found. The information from Table B in S1 Text regarding number of outlier voxels was summarized in Table C in S1 Text, averaged once across map types (left) and once across ROIs (right). The highest percentage of removed voxels was found for the Cerebral-WM (1.4 ± 0.9%) and the T_2_ map (1.8 ± 0.5%), and the lowest for the Putamen (0.8 ± 0.4%) and the ihMTR map (0.5 ± 0.2%). Overall, on average, only a small fraction of ~1.0% of the voxels were labeled as outliers and excluded from the analysis. To assess segmentation consistency across the two types of data (MP2RAGE and synthetic T_1_-weighted data derived by the mrQ software), we compared the volume of each ROI (i.e., volume = “number of voxels” x “voxel resolution”) produced by the two segmentation maps across volunteers. This result is given in the supplementary Table D in S1 Text, providing per ROI the Mean ± SD percentage change in the volume between the two segmented datasets across volunteers. Relative differences higher than 10% were found in the CC (27.2%), putamen (19.0%), Ventral DC (-15.3%) and the entire Cortex (10.9%), suggesting that variability exist and segmentation errors might affect both types of two datasets.

### 3.3 Correlation of qMRI values between right and left hemispheres

**Fig 4:**
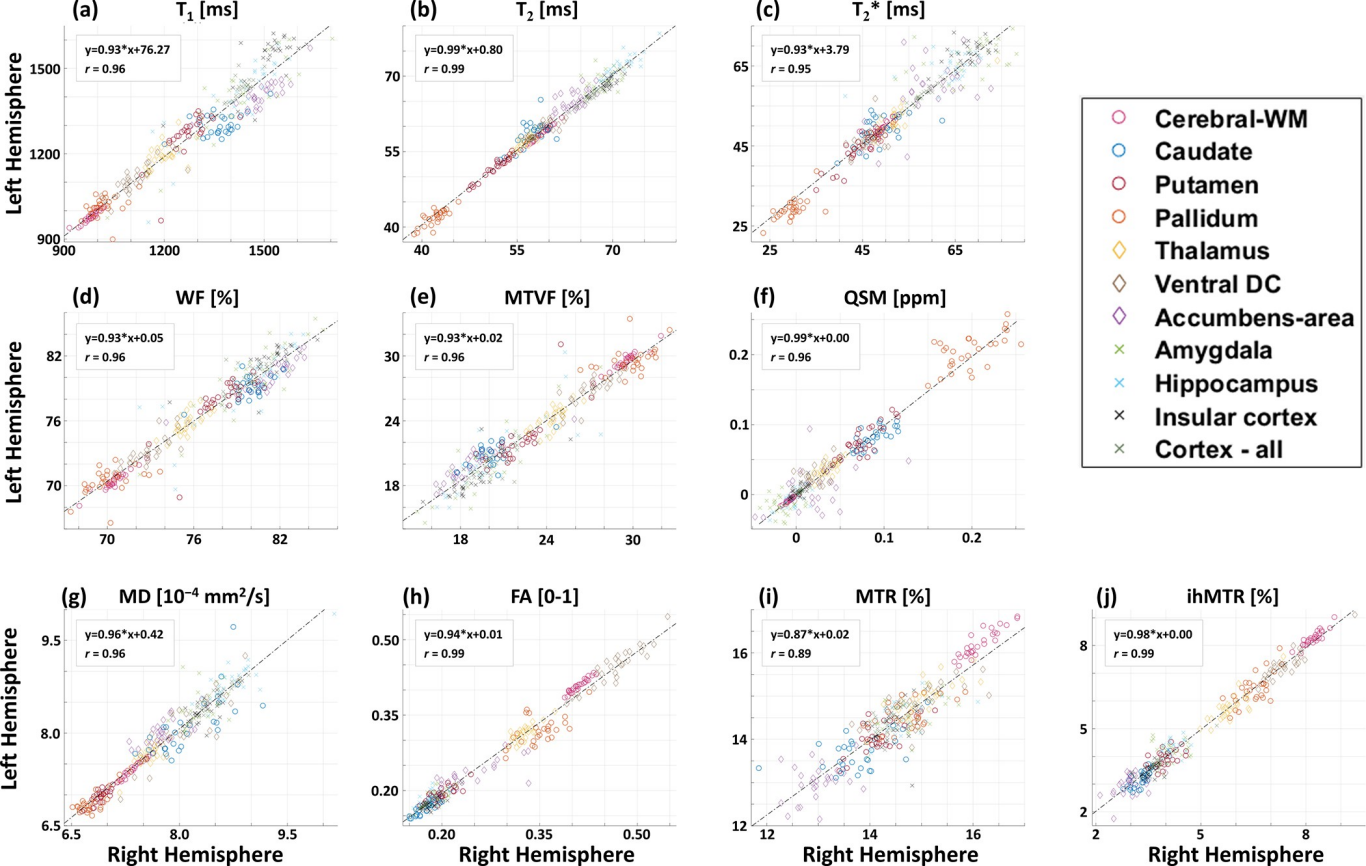
Correlation between quantitative values from the left vs. right hemispheres: **(a)** T_1_, (**b**) T_2_, (**c**) T_2_*, (**d**) WF, (**e**) MTVF, (**f**) QSM, (**g**) MD, (**h**) FA, (**i**) MTR, and (**j**) ihMTR. For each parametric map, the mean values of left versus right hemispheres (total of 28 volunteers) were calculated in 11 representative ROIs (see legend) based on FreeSurfer segmentation. ROIs include cerebral WM, caudate nucleus, putamen, pallidum, thalamus, ventral diencephalon, nucleus accumbens, amygdala, hippocampus, insular cortex, and all-cortex. A linear regression equation is given for each parameter, alongside the Pearson’s correlation coefficient (r). Overall, r > 0.89 represents strong positive relationship between the two hemispheres. *P*-values for all correlations were < 0.001.

Fig 4 shows the correlation of each qMRI parameter between the right and left hemispheres and across all ROIs. Figure is arranged to match the order of the qMRI maps in Fig 3, while each panel presents the linear regression equation (black dotted line), and Pearson’s correlation coefficient *r*.

Mostly linear correlation was observed between the two hemispheres. Some slopes differ from perfect value of 1, with a mean slope of 0.95 ± 0.04 across all maps, reflecting high similarity between right and left hemispheres [86, 87]. Pearson’s correlation coefficients (i.e., *r* scores) were overall higher than 0.95, suggesting a strong and positive relationship between values from left and right hemispheres. One exception was an *r* coefficient of 0.89 for MTR values, reflecting a fairly strong positive relationship. A possible explanation for this weaker trend could be that the left / right differences are influenced by the B_1_^+^ profile, which is typically asymmetric throughout the brain.

The *P* values for all types of parameters were smaller than 0.001, indicating that the linear relationship between the two hemispheres is highly significant. The linear regression was evaluated across all volunteers and all ROIs besides the CC, therefore sample size included 308 values (i.e., N_vol_ x N_ROIs_ = 28 x 11). Some ROIs presented higher right-left variability, e.g., the pallidum in the QSM result, requiring more specific investigation of these differences and their sources.

### 3.4 Scan-rescan analysis

**Fig 5:**
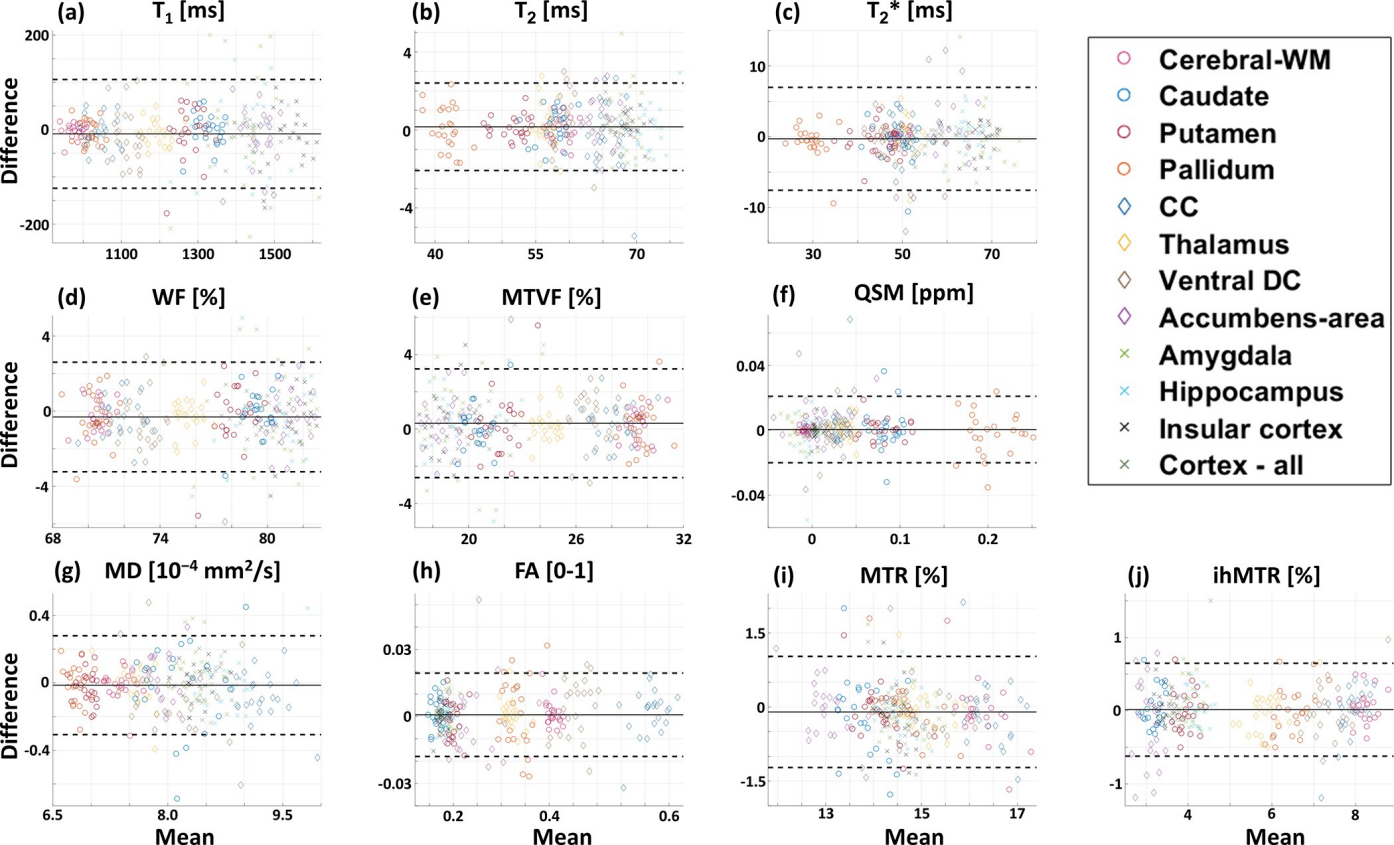
Bland-Altman analysis of scan-rescan stability across qMRI map types. (**a**) T_1_, (**b**) T_2_ (**c**) T_2_*, (**d**) WF, (**e**) MTVF, (**f**) QSM, (**g**) MD, (**h**) FA, (**i**) MTR, and (**j**) ihMTR. For each parametric map, the mean and difference between the two scan sessions were calculated in 12 representative ROIs (see legend): cerebral WM, caudate nucleus, putamen, pallidum, corpus callosum, thalamus, ventral diencephalon, nucleus accumbens, amygdala, hippocampus, insular cortex, and all-cortex.

The scan-rescan data included 23 of the 28 volunteers, who returned for a second scan on a separate day. Fig 5 shows a series of Bland-Altman plots for each map type, where the difference between each two scan sessions is plotted as function of the mean. For each qMRI parameter, Bland-Altman correlation was evaluated across all ROIs, marked with different color and marker shape to a total of 276 comparisons (i.e., N_volunteers_ x N_ROIs_ = 23 x 12). The mean difference and the 95 percent limits of agreement appear as solid and dashed black lines, respectively. Some variability exists between the two scan sessions, although the mean difference across parameters and brain ROIs is close to zero.

Table 4 summarizes the results from the Bland-Altman plots, delineating the mean ± SD of difference between scan sessions, lower and upper limits of agreement (i.e., mean ± 1.96xSD), and the linear regression between the mean of each value (x-axis) and the difference (y-axis) as reflected by the slope, y-intercept, *r* coefficient and the *P*-value. The average and SD of the mean difference were relatively low (i.e., close to zero) for all map types, particularly when considering these values in proportion to the typical dynamic range of each measured parameter, e.g., SD of difference between T_1_ values across two scan sessions was 58.7 ms, while common T_1_ values span a much larger range of ~900–1500 ms.

**Table 4:** Bland-Altman analysis of scan-rescan stability. Properties are shown for each parametric map, corresponding to the Bland-Altman plots in Fig 4. Mean ± SD of difference between the two scan sessions and the 95% limits of agreement were calculated across all 12 ROIs and 23 volunteers. Linear regression between the mean and difference of the two scan sessions showed an overall r = 0.00 ± 0.07 and P = 0.48 ± 0.27, indicating that a poor linear correlation exists between the mean and the difference. Due to the different datasets used for segmentation, i.e., synthetic T_1_w and MP2RAGE images, mrQ related results (namely, T_1_, WF and MTVF maps) are highlighted in grey.

Map type →	T_1_	T_2_	T_2_*	QSM	WF	MTVF	MD	FA	MTR	ihMTR
All ROIs statistics ↓		[ms]	[ms]	[ms]	[ppm]	[%]	[%]	[10^−4^ mm^2^/s]	[0–1]	[%]	[%]
**Mean difference (between sessions)**		-9.0	0.16	-0.29	0.001	-0.31	0.31	-0.01	0.00	-0.10	0.01
**SD of difference**		58.7	1.15	3.71	0.010	1.49	1.49	0.15	0.01	0.57	0.32
95% Limits of Agreement (Mean ±1.96*SD)	**Upper**	106.1	2.40	6.97	0.021	2.61	3.23	0.28	0.02	1.02	0.65
	**Lower**	-124.1	-2.08	-7.56	-0.020	-3.23	-2.61	-0.31	-0.02	-1.23	-0.62
**Linear regression (between mean and difference)**	**slope**	-0.01	0.00	0.01	-0.004	0.01	0.01	-0.01	0.01	-0.09	0.01
	**y-intercept**	7.09	0.22	-0.66	0.001	-0.01	0.00	0.07	0.00	0.01	0.00
	***r* coefficient**	-0.04	-0.01	0.02	-0.03	0.04	0.04	-0.05	0.10	-0.16	0.04
	***P*-value**	0.50	0.91	0.73	0.67	0.51	0.51	0.37	0.08	0.01	0.47

Linear correlation parameters, i.e., the slope and y-intercept, were close to zero as well, indicating a weak trend between the difference and the mean. Overall, *r* coefficients were lower than 0.10, indicating that a poor linear correlation exists between the mean and the difference across the two scan sessions, i.e., similar stability was observed across the physiological range of values for all assayed parameters.

An extension of Table 4 is provided under supplementary Table E in S1 Text, where the Bland-Altman statistics were calculated per ROI (i.e., number of samples per test = number of volunteers = 23). This analysis allows evaluation of scan-rescan reproducibility for a specific pair of map type and ROI; for example, observing the MTR results, a linear correlation between the mean and difference was significant in the Cerbral WM (*P*<0.05), and not significant in the Hippocampus (*P*>0.1).

## 4 Discussion

The clinical applicability of qMRI is continuously increasing, offering a diverse range of numeric biomarkers. This study presents a comprehensive qMRI protocol for brain imaging. The set of pulse-sequences, scan parameters, and postprocessing techniques were all adjusted to produce whole-brain parametric maps at a total scan time of under 50 minutes. The sequences were optimized to accommodate a certain duration and quality, and users can apply different tradeoffs to favor shorter scan times or higher encoding quality based on specific needs. The protocol was applied on 28 healthy volunteers, ages 30–50 y/o, including quantitative evaluation of ten qMRI maps (T_1_, T_2_, T_2_*, WF, MTVF, QSM, MD, FA, MTR, and ihMTR), in twelve brain regions (cerebral-WM, CN, putamen, pallidum, CC, thalamus, ventral diencephalon, nucleus accumbens, amygdala, hippocampus, insular cortex, and all cortical structures), and across two scan sessions. The modularity of the protocol allows users to choose only a subset of pulse-sequences and contrasts of interest, making its use more feasible in clinical settings or for time-limited applications.

The numeric values produced by the presented brain protocol agree with previously published values acquired at 3T [1, 74, 88–91]. It is important to remember that the current study collected data for volunteers in an age range of 30–50 y/o, and in 12 brain ROIs, while values may vary for other populations and brain ROIs as previously reported. Bojorquez et al. [88], for example, compared the relaxation times produced using different qMRI techniques and showed that the range of T_1_ values in the WM is 750–1100 ms across methods, while in our study the corresponding value was 982±22 ms. Gelamn et al. [92] measured the T_2_ values from several WM and gray matter (GM) regions, showing values of 38.8 ± 0.9, 69.4 ± 5.6 and 55.6 ± 0.8 ms for the globus pallidus (GP), prefrontal cortex, and Frontal WM, respectively. These values are similar to our results of 42.0 ± 1.5(GP), 66.4 ± 1.7 (all cortex), and 58.8 ± 1.4 (all cerebral-WM). Treit et al. [90] investigated age related T_2_* changes in healthy individuals across different GM structures. For the relevant comparison group (i.e., 30–50 as in our study), T_2_* values [ms] were- CN: 44.8 ± 1.9, Putamen: 38.2 ± 2.6, Thalamus: 44.5 ± 1.4, and GP: 29.6 ± 6.0, while in our study these were—CN: 48.8 ± 3.5, Putamen: 44.2 ± 3.8, Thalamus: 51.5 ± 4.2, and GP: 29.5 ± 2.4. The higher mean, and SD, of the T_2_* values in some regions, such as the thalamus, suggests that processing method for the T_2_* maps could be improved (i.e., both accuracy and stability), using noise removal or improved signal model. It is also possible that using longer echo times, e.g., up to 70 ms, may improve the fitting accuracy, particularly for tissues with T_2_* > TE_max_. Prolongation of the echo train, however, would come with a price of longer minimal TR, and longer scan times.

For QSM, values across 11 younger adults (21–29 y/o) and 12 elderly adults (64 to 86) were measured by Bilgic et al. [93]. In the younger group values were [10^−2^ ppm]- CN: 9.4 ± 1.9, Putamen: 7.8 ± 1.9, Thalamus: 4.6 ± 2.2, and GP: 12.2 ± 2.5, while in the current study, QSM values were [10^−2^ ppm]- CN: 8.5 ± 1.4, Putamen: 7.7 ± 2.0, Thalamus: 3.7 ± 1.0, and GP: 20.1 ± 2.5. While CN, Putamen, and Thalamus exhibit very high similarity, the difference in mean GP is more significant, and was found to agree with the elderly adults’ group in the study by Bilgic et al, where the average GP value was 19.6 ± 3.2 [10^−2^ ppm]. Importantly, QSM values exhibit variability across various processing methods, which include different approaches for phase unwrapping, background phase removal, and dipole inversion procedures. Multiple techniques are available, and previous studies showed the high variability of the QSM values using different processing steps. For example, Santin et al. [94] compared four QSM processing pipelines, showing a difference of 261% between QSM values in the Red Nucleus, 135% in the Putamen, 40% in the Caudate Nucleus and 44% in the Globus Pallidus. It is agreed that different algorithms may be best suited for different applications [95].

In a study published by Lee et al. [96], the DTI parameters, FA and MD, were measured across 31 individuals in two age groups of 19–39 and 40–65 y/o. For the older group, FA and MD values measured in the Putamen were 0.14 ± 0.03 and 3.0 ± 0.6 [10^−4^ mm^2^/s], and GP values were 0.22 ± 0.07 and 7.3 ± 1.5[10^−4^ mm^2^/s], respectively. In this study values in the Putamen were 0.20 ± 0.01 and 6.9 ± 0.1 [10^−4^ mm^2^/s], and GP values were 0.33 ± 0.03 and 6.8 ± 0.2 [10^−4^ mm^2^/s]. The higher difference in mean MD value of the Putamen between these two studies (i.e., 3.0 vs. 6.9 [10^−4^ mm^2^/s]) can be associated with age, considering the value of 7.1 ± 0.4 10^−4^ mm^2^/s which was collected for the younger group in Lee study. Regarding the tissue content fractions, Meyers et al. [97] calculated the total water content (equivalent to WF in this study) of 10 healthy subjects. Comparing WF values of Meyers et al. vs. the current study [%]: CC: 69.6 ± 0.9 (Splenium) vs 71.9 ± 1.2 (all CC); WM: 71.0 ± 0.5 vs 70.5 ± 0.8; Thalamus: 78.8 ± 1.0 vs 75.7 ± 0.9; and cortex: 84.6 ± 1.6 vs. 79.5 ± 1.1, where the higher difference in the cortex values can be related to the segmentation and calcification processes.

The MTR and ihMTR values are more difficult to compare with previous studies, which acquired the data using different pulse-sequence schemes, due to the dependency of these values on the degree of saturation of the macromolecular pool [7]. MT-related maps in this work were produced by the ihMT protocol developed by Mchinda et al. [8], and our results agree with previous works employing this sequence scheme [77, 98]. An example for alternative approach for MTR calculation, is to reconstruct MT-related maps by repeating a single flip angle 3D-FLASH sequence (Sequence #6) twice, once with, and once without an MT preparation pulse (denoted as MTon and MToff, respectively) [7]. MTR values calculated using this approach are expected to differ from the values generated by the ihMT pipeline, mainly due to differences in the MT saturation pulse properties, e.g., pulse duration, amplitude, and off-resonance frequency, and in the relaxation delays and associated direct water saturation effects [7, 77, 98].

### 4.1 Variability of qMRI values between scan sessions, brain regions, and subjects

Inter-subject variability was almost four times lower than intra-ROI variability, indicating the protocol’s high reproducibility and the relatively high heterogeneity of qMRI parameters within brain segments. The variability between and within brain tissues might originate from thermal noise as well as from intrinsic physiological differences in chemical composition, concentration of metabolites [99], molecular interactions, or hardware instabilities [100]. A possible reason for the lower inter-subject variability can be the high degree of consistency in the basic organization of the brain structures across individuals. The intra-ROI variability on the other hand is expected to be relatively high due to each structure’s specific properties, e.g., the cortex is organized into different layers [101], and the Thalamus is composed of different nuclei [102]. Lastly, one can expect the inter-subject variability to increase if the cohort of volunteers is expanded to include a wider age range [13, 14, 103]. High correlation was found between right and left hemispheres, and while most of the qMRI parameters exhibited *r*>0.95 across ROIs, some variability was observed between the two brain hemispheres. Possible sources for this variability could be neurophysiological differences between the two hemispheres, non-symmetric distributions of the B_0_, B_1_^+^, or B_1_^–^ fields, as well as registration inconsistencies due to imaging artifacts [87, 104]. The neurophysiological differences can be associated with natural brain asymmetry [105], functional role (e.g., different locations of language centers and spatial processing [106, 107]), and differences in blood supply and metabolic activity which may cause variations in the T_1_ and T_2_ relaxation times [105, 108, 109]. Differences in myelination pattern and connectivity between the two hemispheres can also influence the quantitative parameters. For instance, DTI metrics of WM tracts are affected by differences in the density and orientation of the fibers connecting the two hemispheres [110, 111]. Lastly, differences in chemical composition may also contribute to the asymmetry between hemispheres. For example, the concentration of neurotransmitters, such as dopamine or serotonin can vary between the two hemispheres, while these can influence the relaxation times and degree of MT [105, 111–114]. The highest difference between hemispheres in our study was observed in the MTR maps, where the inter-hemispheric correlation was *r* = 0.89. The lower correlation in this case is mainly associated with the ihMT sequence, which was optimized to produce ihMTR, rather than MTR maps, with relatively high immunity to B_1_^+^ inhomogeneities [115]. It is important to note that correction of magnetic fields inhomogeneities (i.e., B_0_, B_1_^-^, B_1_^+^) was applied where possible, as described in the Methods Section.

The repeatability (or scan-rescan stability) is an important aspect of efficient and reliable qMRI-based studies, particularly for longitudinal and multi-center investigations. Not all studies, however, estimate repeatability across multiple brain maps. One example of a study who did perform such analysis is Aye et al. [116], which measured the scan-rescan reliability of MT_SAT_, PD, R_2_* and R_1_ values on a population of 31 healthy subjects in order to increase the relevance of their quantitative protocol for explorative studies in developmental and training-induced plasticity. For that purpose, and similar to our study, the subjects were scanned twice over a rescan interval of about 4 weeks. In our study, the scan-rescan analysis showed that the variability across map types, volunteers, and brain regions was very close to zero, while exhibiting random scatter around zero, and showing no correlation with mean values (Bland Altman plots in Fig 5). Notwithstanding the large sample size used in our tests, the *P* values of the correlation between mean and difference were all larger than 0.05, with an average of 0.48 ± 0.27, suggesting that linear relationship does not explain the data variation, and variations between scans behave in a more random fashion (i.e., random distribution around zero). The only parameter that showed a small correlation between mean and difference was the MTR (*r* = -0.16, *P*<0.05), meaning that higher MTR values were slightly lower in the second scan. This small bias was influenced mainly by the highest MTR values, which were calculated in the Cerebral-WM and CC, where the rescan results were more concentrated near the lower 95% limits of agreement.

Importantly, the statistical analysis of correlation between left and right hemispheres and scan-rescan was applied on the entire brain ROIs per map type. Some factors, such as the spatial location, were therefore averaged-out in this analysis. Users that are interested in investigating these correlations per ROI can download the full set of values from our GitHub and Figshare repositories, where all quantitative values are provided per volunteer, map type, and ROI (see URLs under the data statement).

Variability of qMRI values was not uniform across different map types, where some parameters exhibited more pronounced inter-scan variability, depending on both the quality of raw data and on the postprocessing method. For example, T_2_ values are slightly higher than the T_2_* values (same order of magnitude), whilst the SD of mean difference was more than x2 times higher in the T_2_* scan-rescan results, suggesting lower repeatability of T_2_* values. This can be attributed to the denoising process, which was applied only on the T_2_ data, and to the larger number of time points that were acquired in the MESE sequence (i.e., number of echoes for T_2_ and T_2_* fitting was 12 and 8, respectively). In addition, although both datasets were acquired using GRAPPA acceleration of 2, the T_2_* data employed slice partial Fourier, which reduces SNR and fitting accuracy.

A noteworthy observation relates to the scan-rescan and the inter-subject variabilities. In Table 3, the inter-subject variability is presented as the SD of mean value across volunteers, which was measured independently in each brain ROI. By averaging the SD across ROIs, one can compare the inter-subject and scan-rescan variabilities, where the later was originally averaged across the ROIs as presented in Table 4. Overall, the two variabilities found to be similar, for example, for QSM and T_1_, values of 0.010 vs. 0.013 ppm and 58.7 vs. 59.6 ms were measured for scan-rescan variability vs. inter-subject variability, respectively. For T_2_* and MTR, the scan-rescan variability was found to be higher, while for other parameters the inter-subject variability was higher across ROIs, indicating the high reproducibility of the applied methods. Similar to the current study, some studies indeed focus on estimating the variability of relaxation times and diffusion-MRI between scan sessions, brain regions, and subjects [37, 74, 117, 118], while others investigate this property for less conventional acquisitions such as functional MRI [119] and MR spectroscopy [120].

Lastly, we note that the variability of qMRI values between scan sessions can also source from errors in the segmentation and registration processes. Generally, we registered the segmented maps (i.e., in the MP2RAGE space) to each qMRI map space to achieve the prescribed voxel resolution of the different sequences. Two pipelines differ in that context, these are the mrQ and DWI processing pipelines. For mrQ, qMRI maps were segmented directly in the map space, this required additional run of the FreeSurfer, however it introduced two advantages- (1) simplification of the processing of mrQ output maps, and, (2) saving additional registration step from the MP2RAGE space, which might introduce variability in the ROI labelling due to misregistration and depend on the registration tool being used. While the same segmentation tool, i.e., FreeSurfer, was consistently used, the use of different datasets for segmentation may lead to variations in the ROIs. This limitation was therefore emphasized and statistical comparison of each ROI volume between the two datasets is provided under supplementary Table D in S1 Text. It can be understood from Table E in S1 Text that variability does exist, and segmentation errors might affect both types of datasets. Such segmentation errors will depend on the segmented image’s SNR, CNR, and spatial resolution. The DWI was the only dataset where qMRI values were registered to the T_1_w images space, rather the opposite. To reduce EPI-related artifacts, the DWI data were acquired with a relatively small matrix size (106x106), and therefore interpolation to the anatomical space was applied. This kind of procedure is common for studies in the field, and previously used for evaluation of WM tracts alternation by tumours and reproducibility between different scanners, protocols, and centers [74–76].

### 4.2 Additional qMRI data

The protocol described herein can be used to generate additional quantitative maps not included in this study. These include diffusion-related maps like axial and radial diffusivity (AD and RD) and maps of the diffusion tensor eigenvalues (*λ*_1_, *λ*_2_ and *λ*_3_) [11]. The mrQ pipeline can be used to produce Surface Interaction Rate (**SIR**), which is the T_1_ normalized by the tissue volume (sensitive to changes in molecular composition), B_1_^+^, and B_1_^–^ field maps, and M_0_ (i.e., the Hadamard product of PD by the receiver coil sensitivities). Lastly, the ihMT pipeline can also produce dual saturation MTR maps, termed MTRd, which are based on the *S*^±^ image magnitude and provide an MT contrast that is free from dipolar order contribution, thus directly amenable to the classical qMT binary spin bath model [121].

Sub voxel information can also be extracted from the protocol using advanced processing algorithms. MESE data, for example, can be used to produce fat-water fraction maps using an extension of the EMC algorithm [122]. A more common feature is the myelin content in the brain’s white matter, which can be derived from parameters like T_2_ [5], T_1_ [123], and ihMTR [80], which are known for their high sensitivity to myelin content. For instance, the ratio of T_1_w by T_2_w data [124] has been proposed as a proxy for myelin; the ratio between ihMTR and MTR was suggested to represent a fractional measure of the myelin macromolecular content [80]; and, lastly, using multi-component T_2_, T_1_ or T_2_* to extract the myelin water fraction (**MWF**) maps [125].

### 4.3 Further optimizations and practical tips

The process of optimizing the presented qMRI protocol raised several challenges, and further adjustments can be made for specific applications. These include changes in the scan settings, e.g., voxel resolution, SNR, CNR, the number of b-values or flip angles for MD or T_1_ mapping; and changes to the postprocessing procedures, e.g., additional image corrections, faster processing times, improved registration, and outlier voxels removal. Importantly, when modifying any sequence parameters, one should remember that improvement of data quality is limited in vivo (e.g., by the SAR, scan time, acoustic noise, and possible nerve stimulation). Below, we highlight a few aspects of the protocol design and scan parameters and suggest solutions to potential problems.

One important parameter is the SAR. The highest SAR was measured in the MESE sequence, resulting from the high number of refocusing RF pulses applied within a single TR (i.e., considering the number of echoes and slices, which were 12 and 32, respectively). High SAR requires one to extend scan times or limit the coverage along the slice dimension. The SAR can be reduced by setting the refocusing flip angles to less than 180°, as was done in this study, and which is accounted for by the EMC algorithm so no loss of accuracy entails [41, 43]. Another way to reduce the SAR is to shorten the ETL. This, however, is less favorable since it reduces the maximal TE below 120 ms (in the current study maximal TE was set to 144 ms), which reduces the T_2_ encoding quality in the white and gray matter tissues (see reference [43] for further details). Regardless, to minimize SAR burden on subjects undergoing the entire scan protocol, we recommend arranging the high SAR protocols, e.g., MESE and ihMT, as far apart from each other as possible.

Other optimization aspects of the MESE sequence include ETL, image resolution, scan duration, and longitudinal coverage (i.e., number of scanned slices). The parametric trade-offs in this case are (*i*) improving the T_2_ fitting accuracy by increasing the ETL will increase the SAR; (*ii*) shortening the scan time by reducing the ETL and TR will reduce the fitting accuracy; and (*iii*) increasing coverage on the 3^rd^ dimension (i.e., by increasing the number of slices) will increase the SAR and scan time. Lastly for MESE, natural variability in brain volumes required small FOV adjustments to avoid folding along the PE direction. In order to keep the same pixel size of 1.0x1.0 mm^2^, FOV and matrix size of eleven volunteers were increased from 192x156 to 212x168, leading to a scan time of 8:05 instead of 7:35 minutes.

T_1_ maps were reconstructed in this study using the variable flip angle (VFA) technique [126], which employs FLASH, or spoiled-GRE sequences, and requires at least two flip angles, e.g., 4° and 20°. Fitting accuracy can be improved by increasing the number of flip angles. In this study we prescribed three flip angles equal to 5°, 12° and 27°, resulting in a total scan time of 3 x 2:12 = 6:36 minutes. Apart from the flip angles, the TE value of the FLASH protocol is also of importance when mapping the T_1_ values. Mathematically, the FLASH signal equation depends on both T_1_ and T_2_* [57]. The first TE in the T_1_ encoding sequence (sequence #6) was set to the minimal possible value (i.e., 3.23 ms), therefore minimizing the T_2_* weighting on the PD maps. If the first TE being used is longer, additional correction should be employed based on the calculated T_2_* values, as suggested by Tabelow et al. [34]. This type of correction is more relevant in regions with high iron content or rich in blood vessels.

The second protocol used for T_1_ mapping, and specifically for B_1_^+^ inhomogeneity correction, is the IR-EPI sequence (Sequence #5), where data were collected at four different TIs = 200, 400, 1200, and 2400 ms, resulting in total scan time of 4 x 1:05 = 4:20 minutes. Users should keep in mind that the TR value chosen for the entire TI-series should be set according to the highest TI value to avoid variability in the image intensity across different TIs. Another consideration of the chosen TR relates to the number of concatenations (i.e., acquiring the slices in series of separate sequence repetitions). Higher number of concatenations reduces contamination due to imperfect slice profiles and inter-slice cross-talk [127], and allows to decrease the minimal TR (i.e., necessary to avoid significant increase in total scan time). To avoid fitting errors due to over-shortening of the TR value, we recommend scanning the data using 5–8 concatenations while keeping the TR above 3 sec. In addition, to improve the IR-EPI data quality, we recommend using fat suppression to reduce chemical shift artifacts.

In this study, the 3D FLASH sequence was used twice: once to reconstruct the T_1_ maps (single-echo acquisition, Sequence #6) and once to calculate T_2_* maps (multi-echo acquisition, Sequence #4). It is possible to merge the two sequences into a single scan [128], in which multi-echo measurements for several flip angles will allow to reduce noise by averaging multiple T_2_* and QSM maps across different flip-angles. In this study we chose to acquire the two FLASH data-sets separately, offering a more modular protocol design, which allows to map specific parameters at a minimal scan time, i.e., investigators who are interested in only T_2_* or QSM maps are not required to repeat the FLASH sequence for different flip angles. This separation also enables to increase the scan time and use it to improve the T_2_* (and QSM) fitting accuracy by increasing the ETL or removing the slice partial Fourier. An additional important aspect of mapping T_2_* values is the range of echo times, which was chosen in this study to be 5.2–36.9 ms. This range was chosen in this study to capture the bulk of the decay pattern for most white / gray matter tissues [90, 129], while trading off the encoding quality of tissues with longer T_2_* like the CSF where values can reach ~350 ms [130]. We note that a larger maximal TE can improve the T_2_* mapping accuracy in some ROIs, while this range of TEs was chosen in order to keep the scan time as low as possible, (seeing as the maximal TE limits the minimal achievable TR).

The recommended scan settings for the ihMT sequence are provided by the developers, which optimized the readout module parameters and MT-preparation to achieve the desired ihMT contrast. The single modification we applied was the slice orientation, where we acquired coronal slices using slab-selective excitation instead of sagittal slices and non-selective excitation. This allowed us to decrease the total scan time by ~1 minute. In general, users must verify that the FOV covers the head in both phase- and slice- encoding directions to avoid aliasing.

Analysis of quantitative values in this work was based on automatic brain segmentation performed using the FreeSurfer tool on T_1_w images. The quality of segmentation depends on the imaged SNR and might be reduced in the presence of motion. As a result, in some cases, specific brain regions were partially or wrongly labeled (e.g., several CC segments were labeled as cerebral-WM). Data from one of the volunteers who repeated the scan for the second time, was excluded from the study, since FreeSurfer failed to perform full brain segmentation, even when employing control points to fix the intensity normalization (i.e., manual selection of voxels in the WM boundaries).

Advanced segmentation techniques are available for specific maps and applications. For example, MRIcloud segmentation [131] is optimized for QSM and can extract deep-brain GM regions that stand out exclusively in susceptibility maps and are not segmented by FreeSurfer. Future work could evaluate the use such segmentation techniques to evaluate whether the variability results hold within regions such as the red nucleus, substantia nigra, or dentate nucleus.

The abovementioned optimizations techniques emphasize the importance of adopting specific approaches for improving data quality. Additional important considerations relate to the scanner’s hardware specifications. These vary between vendors, field strengths, types of gradient and RF coils, and other hardware settings. To address these aspects in a qMRI studies, it is recommended to evaluate the scanner’s accuracy and repeatability prior to scanning large cohorts, including comparison of quantitative values with literature, and evaluating intra-scanner and inter-subject repeatability.

### 4.4 Multiparametric brain mapping

qMRI data can also be collected using pulse-sequences that encode multiple parameters in a single scan, simultaneously producing multiple quantitative maps. Such an approach was introduced in 2008 by Weiskopf and Helmes [132], who introduced a 20 minute multi-echo 3D FLASH protocol for mapping PD, T_1_, MT and T_2_*. A similar approach was later taken by several groups that extended the MP2RAGE sequence to include multi-echo acquisitions [133, 134]. Another approach using GRE data is STrategically Acquired Gradient Echo (STAGE) imaging [135]. This technique has a scan time of ~4–5 min and provides PD, T_1_, T_2_* and QSM maps, along with WM, GM, and CSF segmentation. A conceptually different approach for multiparametric mapping at a single-scan is the MRF technique, which is based on incoherent sampling of the multidimensional parametric space and has a scan time of ~5–6 minutes for full brain coverage while allowing the reconstruction of T_1_, T_2_, PD, and MD maps [136, 137]. Although the tradeoff between time and accuracy is yet to be determined, these protocols have shown promising results, and they can supplant some of the pulse-sequences in the protocol presented herein.

To increase the accessibility of qMRI to the community, open-source toolboxes were developed and publicly published. The hMRI toolbox [34], for example, includes a pipeline for mapping T_1_, T_2_*, PD and MTsat. Another example is the qMRLab toolbox [138], which offers model-based signal-fitting, simulations, and sequence optimization, for various quantitative models. This toolbox contains scripts for mapping of QSM, T_1_, MTVF, T_2_, MTR, MTsat and FA, along with B_0_ and B_1_^+^ fields inhomogeneity and noise maps. To facilitate the use of the qMRI protocol presented herein all processing pipelines used in the study are also available online and can be downloaded directly from the URLs provided in the Data and code availability statements Section.

### 4.5 Significance and future work

The improved sensitivity and reproducibility of qMRI protocols can be utilized for many applications. Employing quantitative MRI parameters as clinical biomarkers would greatly benefit from across-the-board standardization. Statistical analysis in this study did not include evaluations based on age or sex, which can be useful but not feasible using the relatively narrow age range (30–50 y/o) and small cohort used in this study (statistical trends were not detected between age and quantitative values—result not shown). Previous reports support this finding, for example, QSM and T_2_* values in the pallidum and caudate nucleus were found to increase in childhood and reached a plateau in the late 30’s [90].

Multiparametric protocols such as the one suggested herein can be used to generate quantitative brain atlases–either for healthy populations or targeted at specific diseases. For clinical applications, such atlases fall well into the realm of radiomics and enable data-rich diagnosis procedures that consolidate multiple quantitative metrics, and spatially-global data from the entire organ in question. It is expected that such multiparametric approach will reveal information that is hidden when using each metric separately [33], and improve the interpretability of biological processes [139].

## Supporting information

S1 Fig Sample Diffusion-weighted images (DWI) with b-value = 1000 [s/mm^2^] for a single volunteer (F, 31 y/o). Images were acquired using two phase-encoding directions: (**a**) anterior-to-posterior and (**b**) posterior-to-anterior. These images correspond to the DWI images in Fig 2H and 2I, which were acquired using b = 0.
(TIF)

S2 Fig Boxplots of ten MR parameters in twelve representative brain regions: Cerebral-white-matter (WM), caudate nucleus (CN), putamen, pallidum (i.e., the globus pallidus), corpus callosum (CC), thalamus, ventral diencephalon (ventral DC), accumbens area (i.e., nucleus accumbens), amygdala, hippocampus, insular cortex, and cortex-all (i.e., all cortical structures). MR parameters are: (**a**) T_1_, (**b**) T_2_, (**c**) T_2_*, (**d**) QSM, (**e**) WF, (**f**) MTVF, (**g**) MD, (**h**) FA, (**i**) MTR, and (**j**) ihMTR. The red lines denote the sample’s median across the 28 volunteers, and the red cross marks the outliers (i.e., observations beyond the whisker length). This figure is based on the metadata used to generate Table 3.
(TIF)

S1 Text Supplementary tables. (**A**) Quantitative values of ten MR parameters in eleven representative Brain regions, separated into left and right hemispheres. (**B**) Change in the number of voxels and mean qMRI values due to outlier voxel removal, per map type and ROI. (**C**) Percentage of excluded voxels, per map type and ROI. (**D**) Evaluation of segmentation consistency across volunteers. (**E**) Bland-Altman analysis of scan-rescan stability per ROI.
(DOCX)

## Abbreviations

qMRI: quantitative MRI
PD: proton density
MT: magnetization transfer
MTR: MT ratio
ihMTR: inhomogeneous MTR
MTsat: MT saturation
QSM: quantitative susceptibility map
MD: mean diffusivity
FA: fractional anisotropy
WF: water fraction
MTVF: macromolecular tissue volume fraction
MESE: multi-echo spin-echo
EMC: echo modulation curve
IR: inversion recovery
EPI: echo-planner imaging
SAR: specific absorption rate
ETL: echo train length
ROI: region of interest
WM: white matter
CN: caudate nucleus
CC: corpus callosum
DC: diencephalon
