# Supplementary figures and images for: A comprehensive protocol for quantitative magnetic resonance imaging of the brain at 3 Tesla
Source: PLoS One. 2024 May 31;19(5):e0297244. doi: 10.1371/journal.pone.0297244 (PMC11142522; doi:10.1371/journal.pone.0297244)

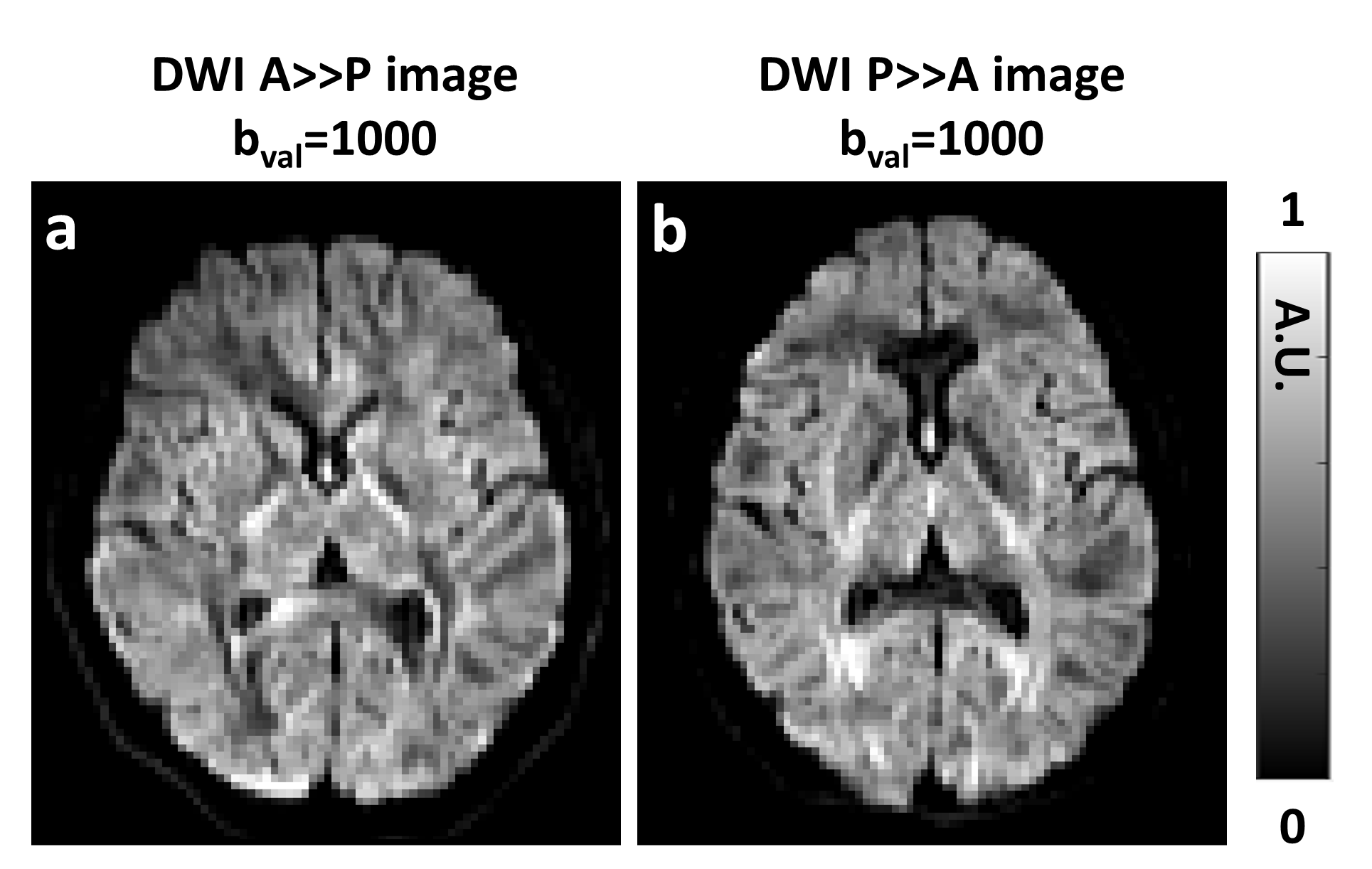

Supplement: S1 Fig — Images were acquired using two phase-encoding directions: (a) anterior-to-posterior and (b) posterior-to-anterior. These images correspond to the DWI images in Fig 2H and 2I, which were acquired using b = 0. (TIF) [file pone.0297244.s001.tif]

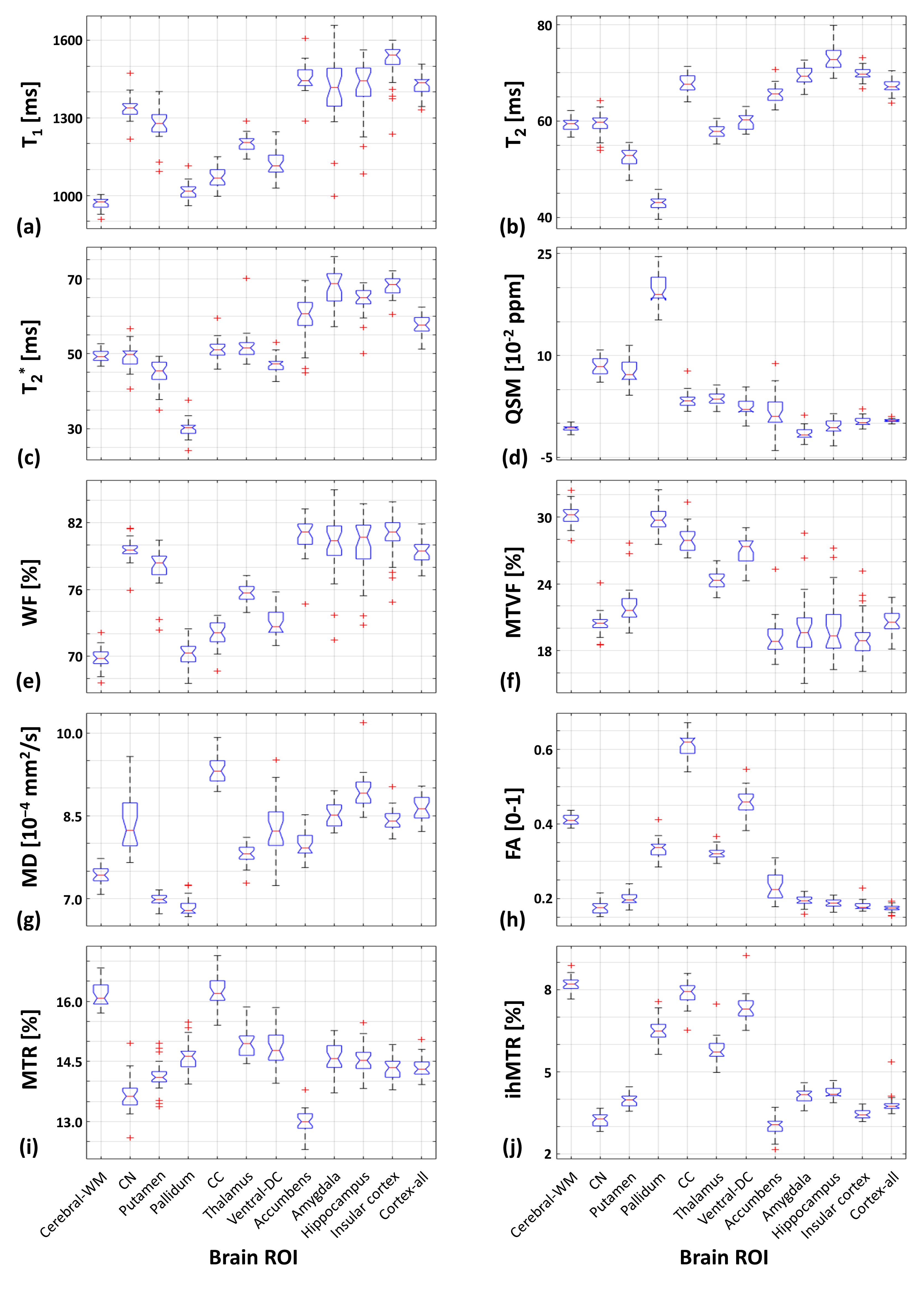

Supplement: S2 Fig — MR parameters are: (a) T1, (b) T2, (c) T2*, (d) QSM, (e) WF, (f) MTVF, (g) MD, (h) FA, (i) MTR, and (j) ihMTR. The red lines denote the sample’s median across the 28 volunteers, and the red cross marks the outliers (i.e., observations beyond the whisker length). This figure is based on the metadata used to generate Table 3. (TIF) [file pone.0297244.s002.tif]
