# Supplementary material for: A comprehensive protocol for quantitative magnetic resonance imaging of the brain at 3 Tesla
Source: PLoS One. 2024 May 31;19(5):e0297244. doi: 10.1371/journal.pone.0297244 (PMC11142522; doi:10.1371/journal.pone.0297244)
Supplement: S1 Text — (A) Quantitative values of ten MR parameters in eleven representative Brain regions, separated into left and right hemispheres. (B) Change in the number of voxels and mean qMRI values due to outlier voxel removal, per map type and ROI. (C) Percentage of excluded voxels, per map type and ROI. (D) Evaluation of segmentation consistency across volunteers. (E) Bland-Altman analysis of scan-rescan stability per ROI. (DOCX) [file pone.0297244.s003.docx]

**A comprehensive protocol for quantitative magnetic resonance imaging of the brain at 3 Tesla**

# Supporting Information file S3 - Supplementary Tables

### **Table A. Quantitative values of ten MR parameters in eleven representative Brain regions, separated into left and right hemispheres:** cerebral-white-matter (WM), caudate nucleus (CN), putamen, pallidum (i.e., the globus pallidus), thalamus, ventral diencephalon (ventral DC), accumbens area (i.e., nucleus accumbens), amygdala, hippocampus, insular cortex, and cortex-all (i.e., all cortical structures). Across the 28 volunteers, values are presented for each MR parameter and ROI. The columns represent: mean ± SD number of voxels, mean ± SD of quantitative value (SD represents the inter-subject variability), and the average SD within the ROI (SD represent the intra-ROI variability). The mean ± SD number of voxels can be translated into volume in cubic millimeters of each ROI, by multiplying these values by the voxel volume of each quantitative map (see Table 1). Due to the different datasets used for segmentation, i.e., synthetic T_1_w and MP2RAGE images, mrQ related results (namely, T_1_, WF and MTVF maps) are highlighted in grey.

| **Map type** | **ROI name** | ***Left Hemisphere*** | | | | | ***Right Hemisphere*** | | | | |  |
| --- | --- | --- | --- | --- | --- | --- | --- | --- | --- | --- | --- | --- |
|  |  | **Number of voxels** | | **Mean qMRI values across volunteers** | | **Intra-ROI variability** | **Number of voxels** | | **Mean qMRI values across volunteers** | | **Intra-ROI variability** |  |
|  |  |  |  |  |  |  |  |  |  |  |  |  |
|  |  | **Mean** | **SD** | **Mean** | **SD** |  | **Mean** | **SD** | **Mean** | **SD** |  |  |
| ***T_1_*** |  | [#] | [#] | [ms] | [ms] | [ms] | [#] | [#] | [ms] | [ms] | [ms] |  |
|  | Cerebral-WM | 92179 | 11973 | 982 | 23 | 143 | 90737 | 12078 | 982 | 21 | 144 |  |
|  | Caudate Nucleous | 945 | 117 | 1367 | 59 | 109 | 901 | 113 | 1302 | 38 | 97 |  |
|  | Putamen | 1231 | 271 | 1275 | 56 | 110 | 1162 | 329 | 1269 | 76 | 102 |  |
|  | Pallidum | 497 | 151 | 1015 | 38 | 68 | 511 | 154 | 1014 | 35 | 64 |  |
|  | Thalamus | 2760 | 551 | 1196 | 38 | 156 | 2739 | 565 | 1193 | 41 | 142 |  |
|  | VentralDC | 1754 | 371 | 1112 | 56 | 195 | 1675 | 376 | 1110 | 49 | 186 |  |
|  | Accumbens-area | 164 | 43 | 1493 | 62 | 112 | 132 | 41 | 1409 | 50 | 123 |  |
|  | Amygdala | 505 | 129 | 1422 | 137 | 199 | 572 | 163 | 1391 | 143 | 185 |  |
|  | Hippocampus | 1395 | 498 | 1418 | 119 | 211 | 1402 | 520 | 1399 | 139 | 205 |  |
|  | ctx_insula | 1600 | 291 | 1512 | 82 | 193 | 1467 | 201 | 1537 | 88 | 204 |  |
|  | ctx | 46081 | 5324 | 1423 | 40 | 296 | 45589 | 6081 | 1423 | 44 | 282 |  |
|  |  |  |  |  |  |  |  |  |  |  |  |  |
| ***T_2_*** |  | [#] | [#] | [ms] | [ms] | [ms] | [#] | [#] | [ms] | [ms] | [ms] |  |
|  | Cerebral-WM | 60989 | 7438 | 58.9 | 1.3 | 4.8 | 60516 | 7661 | 58.7 | 1.4 | 4.8 |  |
|  | Caudate Nucleous | 891 | 99 | 57.3 | 2.0 | 7.0 | 911 | 99 | 58.5 | 2.4 | 7.9 |  |
|  | Putamen | 1385 | 146 | 51.8 | 2.2 | 4.5 | 1365 | 129 | 52.1 | 2.3 | 4.5 |  |
|  | Pallidum | 486 | 73 | 42.1 | 1.6 | 6.5 | 477 | 68 | 41.9 | 1.6 | 5.9 |  |
|  | Thalamus | 2146 | 212 | 56.5 | 1.3 | 5.8 | 2059 | 225 | 57.1 | 1.3 | 6.2 |  |
|  | VentralDC | 1047 | 115 | 59.0 | 1.9 | 10.7 | 1069 | 118 | 59.4 | 1.5 | 10.9 |  |
|  | Accumbens-area | 131 | 19 | 64.0 | 1.9 | 5.5 | 130 | 24 | 65.2 | 1.9 | 6.1 |  |
|  | Amygdala | 343 | 50 | 68.6 | 2.0 | 5.4 | 348 | 65 | 69.0 | 2.0 | 5.4 |  |
|  | Hippocampus | 923 | 95 | 71.3 | 2.4 | 8.2 | 932 | 121 | 72.0 | 2.3 | 8.7 |  |
|  | ctx_insula | 1389 | 187 | 69.4 | 1.3 | 7.1 | 1330 | 150 | 68.8 | 1.4 | 6.5 |  |
|  | ctx | 35412 | 3341 | 66.6 | 1.7 | 8.6 | 34483 | 3293 | 66.1 | 1.7 | 8.9 |  |
|  |  |  |  |  |  |  |  |  |  |  |  |  |
| ***T_2_**** |  | [#] | [#] | [ms] | [ms] | [ms] | [#] | [#] | [ms] | [ms] | [ms] |  |
|  | Cerebral-WM | 183896 | 23279 | 49.2 | 1.7 | 7.3 | 183045 | 23501 | 49.3 | 1.9 | 7.2 |  |
|  | Caudate Nucleous | 2656 | 315 | 48.7 | 4.4 | 10.6 | 2727 | 296 | 49.0 | 3.5 | 10.9 |  |
|  | Putamen | 4137 | 433 | 44.1 | 3.7 | 9.8 | 4078 | 388 | 44.2 | 4.2 | 9.6 |  |
|  | Pallidum | 1470 | 223 | 29.7 | 2.7 | 8.1 | 1439 | 192 | 29.2 | 2.6 | 6.8 |  |
|  | Thalamus | 6316 | 625 | 51.7 | 4.8 | 10.4 | 6117 | 599 | 51.2 | 3.8 | 10.4 |  |
|  | VentralDC | 3080 | 298 | 46.2 | 2.5 | 15.8 | 3160 | 319 | 46.5 | 2.8 | 15.8 |  |
|  | Accumbens-area | 380 | 51 | 59.4 | 7.0 | 14.6 | 384 | 56 | 58.8 | 7.5 | 13.7 |  |
|  | Amygdala | 996 | 146 | 68.2 | 7.2 | 17.7 | 1031 | 138 | 66.6 | 4.6 | 16.4 |  |
|  | Hippocampus | 2720 | 251 | 64.1 | 5.8 | 19.2 | 2766 | 310 | 64.7 | 3.3 | 18.9 |  |
|  | ctx_insula | 3948 | 518 | 67.8 | 3.4 | 17.0 | 3808 | 452 | 68.2 | 2.8 | 16.6 |  |
|  | ctx | 110483 | 9503 | 57.7 | 2.9 | 15.6 | 108531 | 8745 | 57.4 | 2.6 | 15.5 |  |
|  |  |  |  |  |  |  |  |  |  |  |  |  |
| ***QSM*** |  | [#] | [#] | [10^-2^ ppm] | [10^-2^ ppm] | [10^-2^ ppm] | [#] | [#] | [10^-2^ ppm] | [10^-2^ ppm] | [10^-2^ ppm] |  |
|  | Cerebral-WM | 183195 | 23879 | -0.76 | 0.41 | 3.7 | 182621 | 24322 | -0.76 | 0.38 | 3.7 |  |
|  | Caudate Nucleous | 2126 | 315 | 8.85 | 1.57 | 3.4 | 2204 | 288 | 8.16 | 1.45 | 3.4 |  |
|  | Putamen | 3533 | 438 | 7.62 | 1.97 | 5.2 | 3448 | 400 | 7.69 | 2.16 | 5.2 |  |
|  | Pallidum | 1311 | 226 | 19.85 | 2.97 | 7.4 | 1268 | 201 | 20.50 | 2.50 | 6.6 |  |
|  | Thalamus | 6326 | 679 | 3.60 | 1.01 | 4.2 | 6043 | 668 | 3.74 | 1.03 | 4.2 |  |
|  | VentralDC | 3291 | 381 | 2.23 | 1.41 | 8.9 | 3318 | 359 | 2.72 | 1.52 | 9.0 |  |
|  | Accumbens-area | 339 | 50 | 2.22 | 3.30 | 4.8 | 345 | 70 | 0.81 | 3.25 | 4.5 |  |
|  | Amygdala | 1254 | 125 | -1.49 | 1.33 | 3.5 | 1255 | 168 | -1.53 | 1.31 | 3.6 |  |
|  | Hippocampus | 3135 | 289 | -0.42 | 1.22 | 4.3 | 3196 | 357 | -0.66 | 1.33 | 4.3 |  |
|  | ctx_insula | 3501 | 491 | 0.24 | 0.68 | 3.9 | 3376 | 460 | 0.48 | 0.86 | 3.9 |  |
|  | ctx | 110812 | 10204 | 0.44 | 0.24 | 4.1 | 109096 | 9704 | 0.46 | 0.24 | 4.1 |  |
|  |  |  |  |  |  |  |  |  |  |  |  |  |
| ***WF*** |  | [#] | [#] | [%] | [%] | [%] | [#] | [#] | [%] | [%] | [%] |  |
|  | Cerebral-WM | 92160 | 11929 | 70.5 | 0.9 | 4.5 | 90717 | 12102 | 70.5 | 0.8 | 4.5 |  |
|  | Caudate Nucleous | 943 | 117 | 80.0 | 1.3 | 2.2 | 899 | 112 | 79.1 | 1.0 | 2.1 |  |
|  | Putamen | 1226 | 270 | 78.1 | 1.7 | 2.6 | 1157 | 329 | 78.0 | 2.2 | 2.3 |  |
|  | Pallidum | 497 | 152 | 70.2 | 1.4 | 2.6 | 513 | 155 | 70.4 | 1.2 | 2.6 |  |
|  | Thalamus | 2787 | 557 | 75.6 | 0.9 | 3.8 | 2764 | 567 | 75.8 | 1.0 | 3.5 |  |
|  | VentralDC | 1770 | 374 | 72.9 | 1.3 | 5.2 | 1691 | 381 | 73.2 | 1.3 | 4.9 |  |
|  | Accumbens-area | 165 | 43 | 81.4 | 1.9 | 2.4 | 132 | 40 | 80.3 | 1.5 | 2.7 |  |
|  | Amygdala | 504 | 128 | 80.3 | 2.8 | 4.5 | 573 | 163 | 80.1 | 3.3 | 4.2 |  |
|  | Hippocampus | 1398 | 498 | 80.0 | 3.0 | 4.6 | 1403 | 520 | 79.8 | 3.3 | 4.4 |  |
|  | ctx_insula | 1596 | 285 | 81.1 | 1.9 | 3.7 | 1462 | 200 | 81.4 | 1.7 | 3.7 |  |
|  | ctx | 46020 | 5314 | 79.4 | 1.2 | 10.5 | 45509 | 6068 | 79.7 | 1.3 | 9.4 |  |
|  |  |  |  |  |  |  |  |  |  |  |  |  |
| ***MTVF*** |  | [#] | [#] | [%] | [%] | [%] | [#] | [#] | [%] | [%] | [%] |  |
|  | Cerebral-WM | 92160 | 11929 | 29.5 | 0.9 | 4.5 | 90715 | 12102 | 29.5 | 0.8 | 4.5 |  |
|  | Caudate Nucleous | 943 | 117 | 20.0 | 1.3 | 2.2 | 899 | 112 | 20.9 | 1.0 | 2.1 |  |
|  | Putamen | 1226 | 270 | 21.9 | 1.7 | 2.6 | 1157 | 329 | 22.0 | 2.2 | 2.3 |  |
|  | Pallidum | 497 | 152 | 29.8 | 1.4 | 2.6 | 513 | 155 | 29.6 | 1.2 | 2.6 |  |
|  | Thalamus | 2787 | 557 | 24.4 | 0.9 | 3.8 | 2764 | 567 | 24.2 | 1.0 | 3.5 |  |
|  | VentralDC | 1770 | 374 | 27.1 | 1.3 | 5.2 | 1691 | 381 | 26.8 | 1.3 | 4.9 |  |
|  | Accumbens-area | 165 | 43 | 18.6 | 1.9 | 2.4 | 132 | 40 | 19.7 | 1.5 | 2.7 |  |
|  | Amygdala | 504 | 128 | 19.7 | 2.8 | 4.5 | 573 | 163 | 19.9 | 3.3 | 4.2 |  |
|  | Hippocampus | 1398 | 498 | 20.0 | 3.0 | 4.6 | 1403 | 520 | 20.2 | 3.3 | 4.4 |  |
|  | ctx_insula | 1595 | 285 | 18.9 | 1.9 | 3.7 | 1462 | 200 | 18.6 | 1.7 | 3.7 |  |
|  | ctx | 46006 | 5309 | 20.6 | 1.2 | 10.5 | 45494 | 6065 | 20.3 | 1.3 | 9.4 |  |
|  |  |  |  |  |  |  |  |  |  |  |  |  |
| ***MD*** |  | [#] | [#] | [10^-4^ mm^2^/s] | [10^-4^ mm^2^/s] | [10^-4^ mm^2^/s] | [#] | [#] | [10^-4^ mm^2^/s] | [10^-4^ mm^2^/s] | [10^-4^ mm^2^/s] |  |
|  | Cerebral-WM | 182019 | 24025 | 7.34 | 0.15 | 0.5 | 181405 | 24406 | 7.4 | 0.2 | 0.5 |  |
|  | Caudate Nucleous | 2090 | 305 | 8.15 | 0.45 | 2.1 | 2171 | 284 | 8.1 | 0.5 | 1.9 |  |
|  | Putamen | 3485 | 434 | 6.90 | 0.10 | 0.4 | 3396 | 392 | 7.0 | 0.1 | 0.4 |  |
|  | Pallidum | 1315 | 223 | 6.78 | 0.17 | 0.5 | 1272 | 195 | 6.9 | 0.2 | 0.5 |  |
|  | Thalamus | 6191 | 668 | 7.55 | 0.15 | 1.1 | 5908 | 659 | 7.6 | 0.2 | 1.2 |  |
|  | VentralDC | 3222 | 376 | 8.00 | 0.47 | 2.1 | 3244 | 354 | 8.0 | 0.5 | 2.1 |  |
|  | Accumbens-area | 337 | 51 | 7.78 | 0.26 | 0.5 | 339 | 69 | 8.0 | 0.2 | 0.6 |  |
|  | Amygdala | 1234 | 126 | 8.28 | 0.23 | 1.4 | 1234 | 167 | 8.4 | 0.3 | 1.6 |  |
|  | Hippocampus | 3091 | 284 | 8.73 | 0.36 | 1.4 | 3149 | 351 | 8.8 | 0.3 | 1.4 |  |
|  | ctx_insula | 3460 | 487 | 8.33 | 0.20 | 0.9 | 3346 | 450 | 8.3 | 0.2 | 0.9 |  |
|  | ctx | 112654 | 10368 | 8.52 | 0.23 | 1.5 | 110944 | 9650 | 8.6 | 0.2 | 1.5 |  |
|  |  |  |  |  |  |  |  |  |  |  |  |  |
| ***FA*** |  | [#] | [#] | [unitless] | [unitless] | [unitless] | [#] | [#] | [unitless] | [unitless] | [unitless] |  |
|  | Cerebral-WM | 183770 | 24105 | 0.41 | 0.01 | 0.14 | 183169 | 24532 | 0.41 | 0.01 | 0.14 |  |
|  | Caudate Nucleous | 2105 | 309 | 0.18 | 0.02 | 0.06 | 2183 | 288 | 0.17 | 0.01 | 0.06 |  |
|  | Putamen | 3513 | 434 | 0.20 | 0.02 | 0.09 | 3427 | 393 | 0.19 | 0.01 | 0.08 |  |
|  | Pallidum | 1327 | 225 | 0.35 | 0.03 | 0.13 | 1282 | 199 | 0.32 | 0.03 | 0.12 |  |
|  | Thalamus | 6300 | 676 | 0.33 | 0.02 | 0.08 | 6027 | 667 | 0.31 | 0.02 | 0.08 |  |
|  | VentralDC | 3303 | 385 | 0.47 | 0.04 | 0.17 | 3324 | 362 | 0.45 | 0.04 | 0.17 |  |
|  | Accumbens-area | 337 | 50 | 0.23 | 0.05 | 0.07 | 341 | 69 | 0.22 | 0.03 | 0.07 |  |
|  | Amygdala | 1247 | 127 | 0.19 | 0.01 | 0.06 | 1249 | 167 | 0.19 | 0.02 | 0.07 |  |
|  | Hippocampus | 3111 | 283 | 0.18 | 0.01 | 0.07 | 3171 | 351 | 0.18 | 0.01 | 0.07 |  |
|  | ctx_insula | 3488 | 492 | 0.18 | 0.01 | 0.06 | 3373 | 456 | 0.17 | 0.01 | 0.06 |  |
|  | ctx | 113122 | 10490 | 0.17 | 0.01 | 0.07 | 111371 | 9729 | 0.17 | 0.01 | 0.07 |  |
|  |  |  |  |  |  |  |  |  |  |  |  |  |
| ***MTR*** |  | [#] | [#] | [%] | [%] | [%] | [#] | [#] | [%] | [%] | [%] |  |
|  | Cerebral-WM | 183611 | 24358 | 16.1 | 0.3 | 0.9 | 183263 | 24818 | 16.2 | 0.3 | 1.0 |  |
|  | Caudate Nucleous | 2119 | 314 | 13.8 | 0.6 | 0.9 | 2206 | 292 | 13.7 | 0.4 | 0.9 |  |
|  | Putamen | 3523 | 440 | 14.2 | 0.4 | 0.7 | 3424 | 394 | 14.0 | 0.4 | 0.6 |  |
|  | Pallidum | 1329 | 223 | 14.7 | 0.5 | 0.7 | 1279 | 200 | 14.5 | 0.3 | 0.5 |  |
|  | Thalamus | 6244 | 683 | 15.1 | 0.4 | 0.7 | 5957 | 670 | 15.0 | 0.4 | 0.7 |  |
|  | VentralDC | 3267 | 389 | 14.9 | 0.6 | 1.2 | 3290 | 372 | 14.8 | 0.5 | 1.1 |  |
|  | Accumbens-area | 339 | 51 | 13.0 | 0.4 | 0.6 | 343 | 70 | 13.0 | 0.4 | 0.6 |  |
|  | Amygdala | 1247 | 127 | 14.8 | 0.5 | 0.8 | 1247 | 169 | 14.5 | 0.4 | 0.9 |  |
|  | Hippocampus | 3139 | 288 | 14.6 | 0.5 | 1.0 | 3198 | 370 | 14.5 | 0.4 | 1.0 |  |
|  | ctx_insula | 3496 | 489 | 14.5 | 0.3 | 1.1 | 3380 | 460 | 14.3 | 0.4 | 1.0 |  |
|  | ctx | 113082 | 10851 | 14.3 | 0.2 | 1.4 | 111258 | 10021 | 14.4 | 0.3 | 1.4 |  |
|  |  |  |  |  |  |  |  |  |  |  |  |  |
| ***ihMTR*** |  | [#] | [#] | [%] | [%] | [%] | [#] | [#] | [%] | [%] | [%] |  |
|  | Cerebral-WM | 183747 | 24262 | 8.2 | 0.3 | 1.4 | 183255 | 24738 | 8.3 | 0.3 | 1.5 |  |
|  | Caudate Nucleous | 2127 | 319 | 3.3 | 0.2 | 0.9 | 2216 | 292 | 3.2 | 0.3 | 0.9 |  |
|  | Putamen | 3522 | 438 | 4.0 | 0.3 | 0.9 | 3435 | 396 | 3.9 | 0.3 | 0.9 |  |
|  | Pallidum | 1322 | 223 | 6.5 | 0.4 | 1.2 | 1280 | 202 | 6.4 | 0.6 | 1.1 |  |
|  | Thalamus | 6350 | 693 | 5.9 | 0.5 | 1.5 | 6059 | 677 | 5.8 | 0.5 | 1.4 |  |
|  | VentralDC | 3298 | 392 | 7.4 | 0.5 | 1.8 | 3324 | 372 | 7.3 | 0.5 | 1.8 |  |
|  | Accumbens-area | 340 | 51 | 3.0 | 0.4 | 0.7 | 344 | 70 | 3.0 | 0.4 | 0.7 |  |
|  | Amygdala | 1255 | 128 | 4.1 | 0.3 | 0.9 | 1255 | 169 | 4.1 | 0.4 | 1.0 |  |
|  | Hippocampus | 3153 | 293 | 4.2 | 0.2 | 1.0 | 3213 | 370 | 4.3 | 0.3 | 1.0 |  |
|  | ctx_insula | 3521 | 499 | 3.5 | 0.2 | 0.9 | 3386 | 472 | 3.4 | 0.2 | 0.9 |  |
|  | ctx | 112895 | 10910 | 3.8 | 0.4 | 1.2 | 111164 | 10065 | 3.8 | 0.3 | 1.2 |  |

### **Table B.** **Change in the number of voxels and mean qMRI values due to outlier voxel removal, per map type and ROI.** qMRI values presented in the paper are reported after performing weak Chauvenet’s criterion for outlier removal in each ROIs (see Methods section). This table provides the percentage of voxels that were removed by the Chauvenet’s criterion, and the effect on mean qMRI values. The mean ± SD number of voxels can be translated into volume in cubic millimeters of each ROI, by multiplying these values by the voxel volume of each quantitative map (see Table 1). Due to the different datasets used for segmentation, i.e., synthetic T_1_w and MP2RAGE images, mrQ related results (namely, T_1_, WF and MTVF maps) are highlighted in grey.

| **Map type** | **ROI name** | **Number of voxels** | | | | | **Mean qMRI values** | | | | |
| --- | --- | --- | --- | --- | --- | --- | --- | --- | --- | --- | --- |
|  |  | **Before outlier removal** | | **After outlier removal** | | **Relative change** | **Before outlier removal** | | **After outlier removal** | | **Relative change** |
|  |  | **Mean** | **SD** | **Mean** | **SD** |  | **Mean** | **SD** | **Mean** | **SD** |  |
| ***T_1_*** |  | [#] | [#] | [#] | [#] | [%] | [ms] | [ms] | [ms] | [ms] | [%] |
|  | Cerebral-WM | 187550 | 24342 | 182915 | 23808 | 2.5 | 969 | 24 | 982 | 22 | -1.3 |
|  | Caudate Nucleous | 1860 | 223 | 1846 | 223 | 0.8 | 1338 | 44 | 1335 | 43 | 0.2 |
|  | Putamen | 2402 | 571 | 2393 | 568 | 0.4 | 1274 | 61 | 1273 | 61 | 0.0 |
|  | Pallidum | 1014 | 293 | 1008 | 291 | 0.6 | 1016 | 31 | 1015 | 31 | 0.1 |
|  | Corpus callusom | 1183 | 150 | 1165 | 147 | 1.5 | 1067 | 41 | 1051 | 42 | 1.5 |
|  | Thalamus | 5567 | 1087 | 5499 | 1076 | 1.3 | 1204 | 37 | 1195 | 35 | 0.7 |
|  | VentralDC | 3495 | 728 | 3430 | 707 | 1.8 | 1119 | 52 | 1110 | 49 | 0.8 |
|  | Accumbens-area | 299 | 74 | 296 | 74 | 1.0 | 1455 | 55 | 1455 | 56 | 0.0 |
|  | Amygdala | 1085 | 280 | 1077 | 277 | 0.7 | 1408 | 139 | 1405 | 137 | 0.2 |
|  | Hippocampus | 2818 | 992 | 2796 | 984 | 0.8 | 1414 | 119 | 1411 | 120 | 0.2 |
|  | ctx_insula | 3111 | 445 | 3067 | 444 | 1.5 | 1515 | 80 | 1524 | 79 | -0.5 |
|  | ctx | 92524 | 11219 | 91671 | 11122 | 0.9 | 1425 | 40 | 1423 | 40 | 0.1 |
|  |  |  |  |  |  |  |  |  |  |  |  |
| ***T_2_*** |  | [#] | [#] | [#] | [#] | [%] | [ms] | [ms] | [ms] | [ms] | [%] |
|  | Cerebral-WM | 122716 | 15187 | 121505 | 15084 | 1.0 | 59.2 | 1.4 | 58.8 | 1.4 | 0.7 |
|  | Caudate Nucleous | 1843 | 200 | 1802 | 195 | 2.3 | 59.4 | 2.2 | 57.9 | 2.0 | 2.5 |
|  | Putamen | 2776 | 271 | 2750 | 271 | 1.0 | 52.3 | 2.2 | 52.0 | 2.2 | 0.6 |
|  | Pallidum | 987 | 138 | 963 | 135 | 2.5 | 42.8 | 1.5 | 42.0 | 1.5 | 1.8 |
|  | Corpus callusom | 938 | 122 | 915 | 120 | 2.5 | 67.7 | 2.2 | 65.7 | 1.9 | 2.9 |
|  | Thalamus | 4288 | 433 | 4205 | 425 | 2.0 | 57.8 | 1.4 | 56.8 | 1.3 | 1.8 |
|  | VentralDC | 2142 | 230 | 2116 | 229 | 1.2 | 59.9 | 1.6 | 59.2 | 1.6 | 1.2 |
|  | Accumbens-area | 267 | 38 | 261 | 38 | 2.2 | 65.6 | 1.9 | 64.6 | 1.7 | 1.5 |
|  | Amygdala | 702 | 112 | 691 | 110 | 1.5 | 69.5 | 1.9 | 68.8 | 1.6 | 1.0 |
|  | Hippocampus | 1890 | 212 | 1855 | 208 | 1.9 | 73.0 | 2.5 | 71.7 | 2.3 | 1.8 |
|  | ctx_insula | 2764 | 331 | 2719 | 326 | 1.6 | 69.9 | 1.4 | 69.1 | 1.3 | 1.1 |
|  | ctx | 71156 | 6627 | 69895 | 6537 | 1.8 | 67.2 | 1.7 | 66.4 | 1.7 | 1.3 |
|  |  |  |  |  |  |  |  |  |  |  |  |
| ***T_2_**** |  | [#] | [#] | [#] | [#] | [%] | [ms] | [ms] | [ms] | [ms] | [%] |
|  | Cerebral-WM | 373015 | 47442 | 366941 | 46739 | 1.6 | 49.4 | 1.6 | 49.2 | 1.7 | 0.3 |
|  | Caudate Nucleous | 5456 | 602 | 5384 | 597 | 1.3 | 49.3 | 3.3 | 48.8 | 3.5 | 1.0 |
|  | Putamen | 8319 | 825 | 8215 | 811 | 1.3 | 44.7 | 3.8 | 44.2 | 3.8 | 1.2 |
|  | Pallidum | 2962 | 407 | 2909 | 403 | 1.8 | 30.1 | 2.4 | 29.5 | 2.4 | 2.1 |
|  | Corpus callusom | 2757 | 371 | 2710 | 365 | 1.7 | 51.2 | 2.7 | 50.5 | 2.8 | 1.3 |
|  | Thalamus | 12611 | 1208 | 12433 | 1182 | 1.4 | 52.0 | 4.1 | 51.5 | 4.2 | 0.9 |
|  | VentralDC | 6311 | 613 | 6240 | 606 | 1.1 | 47.0 | 2.2 | 46.3 | 2.2 | 1.4 |
|  | Accumbens-area | 772 | 98 | 764 | 98 | 1.1 | 59.7 | 6.1 | 59.1 | 6.4 | 0.9 |
|  | Amygdala | 2036 | 256 | 2027 | 255 | 0.4 | 67.5 | 4.9 | 67.4 | 5.0 | 0.2 |
|  | Hippocampus | 5495 | 531 | 5487 | 529 | 0.2 | 64.5 | 3.9 | 64.4 | 4.0 | 0.1 |
|  | ctx_insula | 7787 | 929 | 7756 | 926 | 0.4 | 68.0 | 2.6 | 67.9 | 2.7 | 0.1 |
|  | ctx | 221395 | 18163 | 219014 | 17995 | 1.1 | 57.9 | 2.6 | 57.5 | 2.7 | 0.6 |
|  |  |  |  |  |  |  |  |  |  |  |  |
| ***QSM*** |  | [#] | [#] | [#] | [#] | [%] | [10^-2^ ppm] | [10^-2^ ppm] | [10^-2^ ppm] | [10^-2^ ppm] | [%] |
|  | Cerebral-WM | 367874 | 48491 | 365817 | 48168 | 0.6 | -0.69 | 0.39 | -0.76 | 0.39 | -10.6 |
|  | Caudate Nucleous | 4365 | 599 | 4329 | 593 | 0.8 | 8.41 | 1.38 | 8.49 | 1.40 | -1.0 |
|  | Putamen | 6994 | 827 | 6982 | 826 | 0.2 | 7.65 | 1.98 | 7.65 | 1.97 | 0.0 |
|  | Pallidum | 2612 | 412 | 2579 | 416 | 1.3 | 19.85 | 2.37 | 20.14 | 2.46 | -1.5 |
|  | Corpus callusom | 2040 | 258 | 2026 | 255 | 0.7 | 3.46 | 1.18 | 3.40 | 1.18 | 1.6 |
|  | Thalamus | 12401 | 1319 | 12370 | 1314 | 0.3 | 3.68 | 0.95 | 3.67 | 0.95 | 0.4 |
|  | VentralDC | 6618 | 737 | 6610 | 730 | 0.1 | 2.51 | 1.27 | 2.48 | 1.28 | 1.2 |
|  | Accumbens-area | 687 | 110 | 684 | 109 | 0.5 | 1.60 | 2.85 | 1.53 | 2.83 | 3.8 |
|  | Amygdala | 2524 | 275 | 2509 | 272 | 0.6 | -1.48 | 0.97 | -1.50 | 0.97 | -1.1 |
|  | Hippocampus | 6377 | 627 | 6332 | 629 | 0.7 | -0.51 | 1.16 | -0.55 | 1.17 | -7.8 |
|  | ctx_insula | 6938 | 915 | 6878 | 903 | 0.9 | 0.27 | 0.73 | 0.35 | 0.69 | -31.4 |
|  | ctx | 221538 | 19867 | 219908 | 19698 | 0.7 | 0.44 | 0.22 | 0.45 | 0.23 | -2.4 |
|  |  |  |  |  |  |  |  |  |  |  |  |
| ***WF*** |  | [#] | [#] | [#] | [#] | [%] | [%] | [%] | [%] | [%] | [%] |
|  | Cerebral-WM | 187550 | 24342 | 182878 | 23798 | 2.5 | 70 | 1 | 70 | 1 | -1.0 |
|  | Caudate Nucleous | 1860 | 223 | 1842 | 222 | 1.0 | 80 | 1 | 80 | 1 | -0.1 |
|  | Putamen | 2402 | 571 | 2382 | 567 | 0.8 | 78 | 2 | 78 | 2 | -0.1 |
|  | Pallidum | 1014 | 293 | 1010 | 292 | 0.3 | 70 | 1 | 70 | 1 | 0.0 |
|  | Corpus callusom | 1183 | 150 | 1173 | 148 | 0.9 | 72 | 1 | 72 | 1 | 0.2 |
|  | Thalamus | 5567 | 1087 | 5551 | 1084 | 0.3 | 76 | 1 | 76 | 1 | 0.0 |
|  | VentralDC | 3495 | 728 | 3461 | 716 | 0.9 | 73 | 1 | 73 | 1 | -0.1 |
|  | Accumbens-area | 299 | 74 | 296 | 73 | 0.8 | 81 | 2 | 81 | 2 | -0.1 |
|  | Amygdala | 1085 | 280 | 1077 | 277 | 0.7 | 80 | 3 | 80 | 3 | -0.1 |
|  | Hippocampus | 2818 | 992 | 2801 | 984 | 0.5 | 80 | 3 | 80 | 3 | -0.1 |
|  | ctx_insula | 3111 | 445 | 3058 | 435 | 1.7 | 81 | 2 | 81 | 2 | -0.5 |
|  | ctx | 92524 | 11219 | 91529 | 11105 | 1.1 | 79 | 1 | 80 | 1 | -0.2 |
|  |  |  |  |  |  |  |  |  |  |  |  |
| ***MTVF*** |  | [#] | [#] | [#] | [#] | [%] | [%] | [%] | [%] | [%] | [%] |
|  | Cerebral-WM | 187547 | 24341 | 182875 | 23799 | 2.5 | 30 | 1 | 30 | 1 | 2.2 |
|  | Caudate Nucleous | 1860 | 223 | 1842 | 222 | 1.0 | 20 | 1 | 20 | 1 | 0.3 |
|  | Putamen | 2402 | 571 | 2382 | 567 | 0.8 | 22 | 2 | 22 | 2 | 0.2 |
|  | Pallidum | 1014 | 293 | 1010 | 292 | 0.3 | 30 | 1 | 30 | 1 | 0.0 |
|  | Corpus callusom | 1183 | 150 | 1173 | 148 | 0.8 | 28 | 1 | 28 | 1 | -0.4 |
|  | Thalamus | 5567 | 1087 | 5551 | 1084 | 0.3 | 24 | 1 | 24 | 1 | -0.1 |
|  | VentralDC | 3495 | 728 | 3461 | 716 | 0.9 | 27 | 1 | 27 | 1 | 0.3 |
|  | Accumbens-area | 299 | 74 | 296 | 73 | 0.8 | 19 | 2 | 19 | 2 | 0.3 |
|  | Amygdala | 1085 | 280 | 1077 | 277 | 0.7 | 20 | 3 | 20 | 3 | 0.5 |
|  | Hippocampus | 2817 | 992 | 2801 | 984 | 0.5 | 20 | 3 | 20 | 3 | 0.3 |
|  | ctx_insula | 3111 | 445 | 3058 | 435 | 1.7 | 19 | 2 | 19 | 2 | 2.1 |
|  | ctx | 92481 | 11209 | 91500 | 11095 | 1.1 | 21 | 1 | 20 | 1 | 0.8 |
|  |  |  |  |  |  |  |  |  |  |  |  |
| ***MD*** |  | [#] | [#] | [#] | [#] | [%] | [10^-4^ mm^2^/s] | [10^-4^ mm^2^/s] | [10^-4^ mm^2^/s] | [10^-4^ mm^2^/s] | [%] |
|  | Cerebral-WM | 368790 | 48977 | 363423 | 48393 | 1.5 | 7.43 | 0.15 | 7.37 | 0.15 | 0.8 |
|  | Caudate Nucleous | 4366 | 597 | 4261 | 579 | 2.4 | 8.37 | 0.48 | 8.14 | 0.43 | 2.7 |
|  | Putamen | 6993 | 825 | 6881 | 816 | 1.6 | 6.98 | 0.11 | 6.94 | 0.10 | 0.6 |
|  | Pallidum | 2616 | 413 | 2587 | 410 | 1.1 | 6.85 | 0.16 | 6.83 | 0.15 | 0.3 |
|  | Corpus callusom | 2043 | 256 | 2009 | 247 | 1.6 | 9.36 | 0.29 | 9.23 | 0.29 | 1.4 |
|  | Thalamus | 12409 | 1326 | 12100 | 1295 | 2.5 | 7.80 | 0.18 | 7.59 | 0.16 | 2.7 |
|  | VentralDC | 6628 | 736 | 6467 | 719 | 2.4 | 8.30 | 0.53 | 8.02 | 0.48 | 3.4 |
|  | Accumbens-area | 688 | 110 | 675 | 109 | 1.8 | 7.96 | 0.24 | 7.90 | 0.22 | 0.8 |
|  | Amygdala | 2526 | 278 | 2469 | 271 | 2.3 | 8.52 | 0.22 | 8.35 | 0.21 | 2.0 |
|  | Hippocampus | 6383 | 628 | 6240 | 619 | 2.3 | 8.94 | 0.33 | 8.78 | 0.32 | 1.8 |
|  | ctx_insula | 6938 | 909 | 6806 | 889 | 1.9 | 8.43 | 0.20 | 8.34 | 0.18 | 1.1 |
|  | ctx | 227517 | 20239 | 223597 | 19826 | 1.7 | 8.63 | 0.22 | 8.55 | 0.22 | 0.9 |
|  |  |  |  |  |  |  |  |  |  |  |  |
| ***FA*** |  | [#] | [#] | [#] | [#] | [%] | [unitless] | [unitless] | [unitless] | [unitless] | [%] |
|  | Cerebral-WM | 368790 | 48977 | 366939 | 48601 | 0.5 | 0.41 | 0.01 | 0.41 | 0.01 | 0.5 |
|  | Caudate Nucleous | 4366 | 597 | 4288 | 587 | 1.8 | 0.18 | 0.02 | 0.17 | 0.01 | 2.9 |
|  | Putamen | 6993 | 825 | 6940 | 816 | 0.7 | 0.20 | 0.01 | 0.20 | 0.01 | 1.1 |
|  | Pallidum | 2616 | 413 | 2609 | 415 | 0.3 | 0.34 | 0.03 | 0.33 | 0.03 | 0.4 |
|  | Corpus callusom | 2043 | 256 | 2041 | 255 | 0.1 | 0.61 | 0.03 | 0.61 | 0.03 | -0.1 |
|  | Thalamus | 12409 | 1326 | 12327 | 1314 | 0.7 | 0.32 | 0.02 | 0.32 | 0.01 | 0.6 |
|  | VentralDC | 6628 | 736 | 6627 | 735 | 0.0 | 0.46 | 0.04 | 0.46 | 0.04 | 0.0 |
|  | Accumbens-area | 688 | 110 | 678 | 107 | 1.5 | 0.23 | 0.04 | 0.23 | 0.04 | 1.7 |
|  | Amygdala | 2526 | 278 | 2496 | 271 | 1.2 | 0.19 | 0.01 | 0.19 | 0.01 | 1.5 |
|  | Hippocampus | 6383 | 628 | 6283 | 617 | 1.6 | 0.19 | 0.01 | 0.18 | 0.01 | 2.5 |
|  | ctx_insula | 6938 | 909 | 6861 | 901 | 1.1 | 0.18 | 0.01 | 0.18 | 0.01 | 1.5 |
|  | ctx | 227517 | 20239 | 224493 | 20025 | 1.3 | 0.18 | 0.01 | 0.17 | 0.01 | 2.5 |
|  |  |  |  |  |  |  |  |  |  |  |  |
| ***MTR*** |  | [#] | [#] | [#] | [#] | [%] | [%] | [%] | [%] | [%] | [%] |
|  | Cerebral-WM | 369045 | 49377 | 366874 | 49139 | 0.6 | 16.2 | 0.3 | 16.2 | 0.3 | -0.1 |
|  | Caudate Nucleous | 4371 | 601 | 4325 | 594 | 1.0 | 13.7 | 0.4 | 13.7 | 0.5 | -0.2 |
|  | Putamen | 6998 | 826 | 6947 | 822 | 0.7 | 14.1 | 0.4 | 14.1 | 0.4 | 0.1 |
|  | Pallidum | 2619 | 417 | 2608 | 415 | 0.4 | 14.6 | 0.4 | 14.6 | 0.4 | 0.0 |
|  | Corpus callusom | 2046 | 258 | 2023 | 250 | 1.1 | 16.3 | 0.4 | 16.3 | 0.4 | -0.4 |
|  | Thalamus | 12418 | 1337 | 12200 | 1321 | 1.8 | 15.0 | 0.4 | 15.0 | 0.4 | -0.4 |
|  | VentralDC | 6638 | 760 | 6556 | 750 | 1.2 | 14.8 | 0.5 | 14.9 | 0.5 | -0.4 |
|  | Accumbens-area | 689 | 109 | 682 | 108 | 1.0 | 13.0 | 0.3 | 13.0 | 0.3 | -0.1 |
|  | Amygdala | 2528 | 280 | 2495 | 277 | 1.3 | 14.6 | 0.4 | 14.7 | 0.4 | -0.3 |
|  | Hippocampus | 6394 | 644 | 6337 | 640 | 0.9 | 14.5 | 0.4 | 14.6 | 0.4 | -0.2 |
|  | ctx_insula | 6954 | 922 | 6876 | 905 | 1.1 | 14.3 | 0.3 | 14.4 | 0.3 | -0.3 |
|  | ctx | 227060 | 20806 | 224340 | 20659 | 1.2 | 14.4 | 0.3 | 14.4 | 0.3 | -0.2 |
|  |  |  |  |  |  |  |  |  |  |  |  |
| ***ihMTR*** |  | [#] | [#] | [#] | [#] | [%] | [%] | [%] | [%] | [%] | [%] |
|  | Cerebral-WM | 368953 | 49331 | 367002 | 48965 | 0.5 | 8.2 | 0.3 | 8.2 | 0.3 | -0.3 |
|  | Caudate Nucleous | 4369 | 603 | 4342 | 599 | 0.6 | 3.2 | 0.2 | 3.2 | 0.2 | 0.6 |
|  | Putamen | 6998 | 826 | 6957 | 822 | 0.6 | 4.0 | 0.2 | 4.0 | 0.3 | 0.4 |
|  | Pallidum | 2619 | 417 | 2601 | 417 | 0.7 | 6.5 | 0.4 | 6.5 | 0.5 | 0.4 |
|  | Corpus callusom | 2045 | 257 | 2034 | 254 | 0.5 | 7.9 | 0.4 | 7.9 | 0.4 | -0.3 |
|  | Thalamus | 12418 | 1337 | 12408 | 1338 | 0.1 | 5.8 | 0.5 | 5.8 | 0.5 | 0.0 |
|  | VentralDC | 6635 | 758 | 6622 | 754 | 0.2 | 7.4 | 0.5 | 7.4 | 0.5 | -0.1 |
|  | Accumbens-area | 688 | 110 | 684 | 110 | 0.6 | 3.0 | 0.3 | 3.0 | 0.3 | 0.4 |
|  | Amygdala | 2526 | 280 | 2510 | 276 | 0.6 | 4.1 | 0.3 | 4.1 | 0.3 | 0.4 |
|  | Hippocampus | 6392 | 647 | 6366 | 645 | 0.4 | 4.3 | 0.2 | 4.2 | 0.2 | 0.3 |
|  | ctx_insula | 6940 | 925 | 6907 | 925 | 0.5 | 3.5 | 0.2 | 3.4 | 0.2 | 0.4 |
|  | ctx | 224990 | 20808 | 224059 | 20770 | 0.4 | 3.8 | 0.3 | 3.8 | 0.3 | 0.4 |

### **Table C.** **Percentage of excluded voxels, per map type and ROI.** This table is a summary of Table B, providing the average percentage of voxels that were removed by the Chauvenet’s criterion, averaged once across map types (left) and once across ROIs (right). Due to the different datasets used for segmentation, i.e., synthetic T_1_w and MP2RAGE images, mrQ related results (namely, T_1_, WF and MTVF maps) are highlighted in grey.

| **Analysis per ROI (averaged across maps)** | **Mean** | **SD** |  |  | **Analysis per map (averaged across ROIs)** | **Mean** | **SD** |
| --- | --- | --- | --- | --- | --- | --- | --- |
|  | **[%]** | **[%]** |  |  |  | **[%]** | **[%]** |
| **Cerebral-WM** | 1.4 | 0.9 |  |  | **T_1_** | 1.1 | 0.6 |
| **Caudate Nucleous** | 1.3 | 0.6 |  |  | **T_2_** | 1.8 | 0.5 |
| **Putamen** | 0.8 | 0.4 |  |  | **T_2_*** | 1.1 | 0.5 |
| **Pallidum** | 0.9 | 0.7 |  |  | **QSM** | 0.6 | 0.3 |
| **Corpus callusom** | 1.1 | 0.7 |  |  | **WF** | 1.0 | 0.6 |
| **Thalamus** | 1.0 | 0.8 |  |  | **MTVF** | 1.0 | 0.6 |
| **VentralDC** | 1.0 | 0.8 |  |  | **MD** | 1.9 | 0.4 |
| **Accumbens-area** | 1.1 | 0.5 |  |  | **FA** | 0.9 | 0.6 |
| **Amygdala** | 1.0 | 0.6 |  |  | **MTR** | 1.0 | 0.4 |
| **Hippocampus** | 1.0 | 0.7 |  |  | **ihMTR** | 0.5 | 0.2 |
| **ctx_insula** | 1.2 | 0.5 |  |  |  |  |  |
| **ctx** | 1.1 | 0.4 |  |  |  |  |  |

**Table D.** **Evaluation of segmentation consistency across volunteers**. For each ROI, the table presents the Mean ± SD percentage of change between the segmented volumes of the two data sets (i.e., MP2RAGE and Synthetic-T_1_w). ROI volumes were calculated as the number of voxels in ROI x voxel size, which were 1.0x1.0x1.0 and 1.0x1.0x2.2 mm^3^, for MP2RAGE (sequence #2) and SPGR (sequence #6), respectively. The analysis did not include any type of voxel exclusion.

| **ROI name** |  | **Relative change in segmented volume across volunteers** | |
| --- | --- | --- | --- |
|  |  |  |  |
|  |  | **Mean** | **SD** |
|  |  | [%] | [%] |
| Cerebral-WM |  | -9.0 | 10.5 |
| Caudate Nucleous |  | 5.7 | 5.6 |
| Putamen |  | 19.0 | 15.2 |
| Pallidum |  | 7.7 | 19.0 |
| Corpus callusom |  | -27.2 | 14.6 |
| Thalamus |  | 0.7 | 18.8 |
| VentralDC |  | -15.3 | 22.1 |
| Accumbens-area |  | 5.5 | 20.5 |
| Amygdala |  | 4.3 | 21.5 |
| Hippocampus |  | 1.2 | 29.9 |
| ctx_insula |  | 1.2 | 9.1 |
| ctx |  | 10.9 | 6.5 |

**Table E.** **Bland-Altman analysis of scan-rescan stability per ROI.** Values are shown per ROI for each parametric map, corresponding to the Bland-Altman statistics from Table 4, which represents the average across ROIs. Mean ± SD of difference between the two scan sessions and the 95% limits of agreement were calculated across all 12 ROIs and 23 volunteers. Due to the different datasets used for segmentation (i.e., synthetic T_1_w and MP2RAGE), mrQ related results (namely, T_1_, WF and MTVF maps) are highlighted in grey.

| **ROI ↓** | **Map type →** | | **T_1_** | **T_2_** | **T_2_*** | **QSM** | **WF** | **MTVF** | **MD** | **FA** | **MTR** | **ihMTR** |
| --- | --- | --- | --- | --- | --- | --- | --- | --- | --- | --- | --- | --- |
|  | **Units →** | | **[ms]** | **[ms]** | **[ms]** | **[ppm]** | **[%]** | **[%]** | **[10^-4^ mm^2^/s]** | **[0-1]** | **[%]** | **[%]** |
| **Cerebral-WM** | **Mean difference (between sessions)** | | -1.8 | 0.06 | 0.00 | 0.001 | -0.21 | 0.21 | -0.01 | 0.00 | -0.12 | 0.06 |
|  | **SD of difference** | | 15.0 | 0.65 | 0.93 | 0.003 | 0.82 | 0.82 | 0.08 | 0.01 | 0.61 | 0.22 |
|  | **95% Limits of Agreement (Mean ±1.96*SD)** | **Upper** | 27.6 | 1.33 | 1.82 | 0.006 | 1.40 | 1.83 | 0.15 | 0.01 | 1.08 | 0.50 |
|  |  | **Lower** | -31.3 | -1.21 | -1.82 | -0.004 | -1.83 | -1.40 | -0.17 | -0.01 | -1.31 | -0.37 |
|  | **Linear regression (between mean and difference)** | **slope** | 0.05 | -0.02 | 0.17 | 0.024 | 0.26 | 0.26 | -0.05 | -0.22 | -0.87 | 0.03 |
|  |  | **y-intercept** | -46.20 | 1.45 | -8.18 | 0.001 | -0.19 | -0.08 | 0.39 | 0.09 | 0.14 | 0.00 |
|  |  | ***r* coefficient** | 0.06 | -0.05 | 0.29 | 0.04 | 0.20 | 0.20 | -0.11 | -0.46 | -0.58 | 0.04 |
|  |  | ***P*-value** | 0.78 | 0.81 | 0.17 | 0.86 | 0.37 | 0.37 | 0.63 | 0.03 | 0.00 | 0.87 |
|  |  |  |  |  |  |  |  |  |  |  |  |  |
| **Caudate Nucleous** | **Mean difference (between sessions)** | | 0.0 | 0.26 | -0.60 | 0.001 | -0.10 | 0.10 | -0.02 | 0.00 | -0.17 | 0.04 |
|  | **SD of difference** | | 34.6 | 0.86 | 2.81 | 0.012 | 1.15 | 1.15 | 0.25 | 0.01 | 0.81 | 0.26 |
|  | **95% Limits of Agreement (Mean ±1.96*SD)** | **Upper** | 67.8 | 1.94 | 4.92 | 0.025 | 2.15 | 2.35 | 0.47 | 0.02 | 1.43 | 0.55 |
|  |  | **Lower** | -67.7 | -1.42 | -6.11 | -0.024 | -2.35 | -2.15 | -0.51 | -0.01 | -1.77 | -0.48 |
|  | **Linear regression (between mean and difference)** | **slope** | -0.03 | 0.12 | -0.16 | 0.027 | 0.46 | 0.46 | 0.03 | 0.05 | -0.69 | 0.25 |
|  |  | **y-intercept** | 34.58 | -6.48 | 7.41 | -0.002 | -0.36 | -0.09 | -0.25 | -0.01 | 0.09 | -0.01 |
|  |  | ***r* coefficient** | -0.02 | 0.27 | -0.22 | 0.03 | 0.28 | 0.28 | 0.05 | 0.09 | -0.36 | 0.19 |
|  |  | ***P*-value** | 0.92 | 0.22 | 0.32 | 0.90 | 0.19 | 0.19 | 0.83 | 0.67 | 0.09 | 0.39 |
|  |  |  |  |  |  |  |  |  |  |  |  |  |
| **Putamen** | **Mean difference (between sessions)** | | -5.8 | 0.12 | -0.70 | 0.001 | -0.20 | 0.20 | -0.02 | 0.00 | -0.12 | -0.02 |
|  | **SD of difference** | | 54.5 | 0.56 | 2.16 | 0.006 | 1.65 | 1.65 | 0.10 | 0.01 | 0.52 | 0.28 |
|  | **95% Limits of Agreement (Mean ±1.96*SD)** | **Upper** | 101.0 | 1.22 | 3.55 | 0.013 | 3.02 | 3.43 | 0.17 | 0.01 | 0.89 | 0.53 |
|  |  | **Lower** | -112.5 | -0.98 | -4.94 | -0.011 | -3.43 | -3.02 | -0.22 | -0.02 | -1.13 | -0.57 |
|  | **Linear regression (between mean and difference)** | **slope** | 0.33 | 0.03 | 0.00 | -0.024 | 0.76 | 0.76 | -0.21 | -0.04 | -0.66 | 0.12 |
|  |  | **y-intercept** | -424.51 | -1.35 | -0.69 | 0.003 | -0.60 | -0.16 | 1.41 | 0.01 | 0.09 | 0.00 |
|  |  | ***r* coefficient** | 0.20 | 0.11 | 0.00 | -0.08 | 0.41 | 0.41 | -0.23 | -0.06 | -0.54 | 0.10 |
|  |  | ***P*-value** | 0.36 | 0.60 | 1.00 | 0.72 | 0.05 | 0.05 | 0.29 | 0.77 | 0.01 | 0.66 |
|  |  |  |  |  |  |  |  |  |  |  |  |  |
| **Pallidum** | **Mean difference (between sessions)** | | -5.4 | 0.10 | -0.59 | -0.002 | -0.25 | 0.25 | -0.01 | 0.00 | -0.04 | -0.01 |
|  | **SD of difference** | | 25.2 | 1.11 | 2.33 | 0.014 | 1.28 | 1.28 | 0.11 | 0.02 | 0.53 | 0.32 |
|  | **95% Limits of Agreement (Mean ±1.96*SD)** | **Upper** | 44.0 | 2.29 | 3.98 | 0.024 | 2.26 | 2.77 | 0.21 | 0.03 | 1.00 | 0.63 |
|  |  | **Lower** | -54.8 | -2.08 | -5.16 | -0.029 | -2.77 | -2.26 | -0.23 | -0.03 | -1.08 | -0.65 |
|  | **Linear regression (between mean and difference)** | **slope** | -0.10 | -0.25 | -0.26 | 0.025 | 0.15 | 0.15 | -0.11 | -0.01 | -0.71 | 0.40 |
|  |  | **y-intercept** | 91.80 | 10.55 | 7.22 | -0.007 | -0.11 | -0.04 | 0.77 | 0.00 | 0.10 | -0.03 |
|  |  | ***r* coefficient** | -0.08 | -0.38 | -0.31 | 0.04 | 0.08 | 0.08 | -0.14 | -0.01 | -0.63 | 0.47 |
|  |  | ***P*-value** | 0.70 | 0.07 | 0.15 | 0.84 | 0.71 | 0.71 | 0.54 | 0.95 | 0.00 | 0.02 |
|  |  |  |  |  |  |  |  |  |  |  |  |  |
| **Corpus callusom** | **Mean difference (between sessions)** | | -6.5 | -0.33 | -0.02 | 0.004 | -0.33 | 0.33 | -0.07 | 0.00 | -0.11 | -0.01 |
|  | **SD of difference** | | 32.2 | 1.82 | 2.85 | 0.015 | 1.06 | 1.06 | 0.13 | 0.01 | 0.68 | 0.36 |
|  | **95% Limits of Agreement (Mean ±1.96*SD)** | **Upper** | 56.7 | 3.23 | 5.57 | 0.033 | 1.73 | 2.40 | 0.19 | 0.02 | 1.23 | 0.70 |
|  |  | **Lower** | -69.7 | -3.89 | -5.60 | -0.025 | -2.40 | -1.74 | -0.33 | -0.02 | -1.45 | -0.72 |
|  | **Linear regression (between mean and difference)** | **slope** | -0.06 | -0.18 | -0.04 | 0.679 | 0.08 | 0.07 | -0.13 | 0.12 | -0.62 | 0.72 |
|  |  | **y-intercept** | 55.03 | 11.53 | 1.85 | -0.017 | -0.06 | -0.02 | 1.09 | -0.07 | 0.10 | -0.06 |
|  |  | ***r* coefficient** | -0.07 | -0.18 | -0.03 | 0.34 | 0.08 | 0.07 | -0.26 | 0.35 | -0.37 | 0.63 |
|  |  | ***P*-value** | 0.75 | 0.40 | 0.88 | 0.11 | 0.73 | 0.74 | 0.23 | 0.11 | 0.09 | 0.00 |
|  |  |  |  |  |  |  |  |  |  |  |  |  |
| **Thalamus** | **Mean difference (between sessions)** | | -7.3 | 0.25 | 0.67 | 0.001 | -0.26 | 0.26 | -0.01 | 0.00 | -0.05 | -0.02 |
|  | **SD of difference** | | 25.5 | 1.10 | 5.39 | 0.006 | 0.83 | 0.83 | 0.12 | 0.01 | 0.45 | 0.27 |
|  | **95% Limits of Agreement (Mean ±1.96*SD)** | **Upper** | 42.7 | 2.40 | 11.24 | 0.013 | 1.36 | 1.88 | 0.22 | 0.01 | 0.83 | 0.51 |
|  |  | **Lower** | -57.3 | -1.89 | -9.90 | -0.011 | -1.88 | -1.36 | -0.24 | -0.01 | -0.92 | -0.56 |
|  | **Linear regression (between mean and difference)** | **slope** | -0.08 | -0.14 | 1.23 | -0.070 | 0.17 | 0.17 | -0.33 | -0.15 | -0.39 | 0.30 |
|  |  | **y-intercept** | 86.32 | 8.03 | -62.08 | 0.004 | -0.13 | -0.04 | 2.50 | 0.05 | 0.06 | -0.02 |
|  |  | ***r* coefficient** | -0.10 | -0.15 | 0.56 | -0.11 | 0.13 | 0.13 | -0.53 | -0.37 | -0.35 | 0.46 |
|  |  | ***P*-value** | 0.66 | 0.49 | 0.01 | 0.62 | 0.55 | 0.55 | 0.01 | 0.08 | 0.10 | 0.03 |
|  |  |  |  |  |  |  |  |  |  |  |  |  |
| **VentralDC** | **Mean difference (between sessions)** | | -16.9 | 0.16 | -0.12 | 0.000 | -0.50 | 0.49 | -0.11 | 0.00 | -0.03 | -0.01 |
|  | **SD of difference** | | 58.2 | 1.43 | 2.53 | 0.010 | 1.43 | 1.43 | 0.21 | 0.01 | 0.58 | 0.35 |
|  | **95% Limits of Agreement (Mean ±1.96*SD)** | **Upper** | 97.1 | 2.96 | 4.84 | 0.019 | 2.31 | 3.29 | 0.30 | 0.03 | 1.11 | 0.68 |
|  |  | **Lower** | -130.9 | -2.65 | -5.09 | -0.019 | -3.30 | -2.31 | -0.53 | -0.02 | -1.17 | -0.69 |
|  | **Linear regression (between mean and difference)** | **slope** | -0.33 | 0.05 | -0.38 | 0.274 | 0.18 | 0.18 | -0.13 | 0.11 | -0.36 | 0.30 |
|  |  | **y-intercept** | 344.73 | -2.82 | 17.32 | -0.007 | -0.14 | -0.04 | 0.97 | -0.05 | 0.05 | -0.02 |
|  |  | ***r* coefficient** | -0.17 | 0.05 | -0.39 | 0.32 | 0.10 | 0.10 | -0.29 | 0.28 | -0.34 | 0.39 |
|  |  | ***P*-value** | 0.43 | 0.80 | 0.07 | 0.14 | 0.64 | 0.65 | 0.18 | 0.19 | 0.12 | 0.07 |
|  |  |  |  |  |  |  |  |  |  |  |  |  |
| **Accumbens-area** | **Mean difference (between sessions)** | | -18.9 | 0.46 | 0.05 | 0.004 | -0.51 | 0.51 | 0.03 | 0.00 | -0.11 | -0.17 |
|  | **SD of difference** | | 62.6 | 1.24 | 6.22 | 0.017 | 1.77 | 1.77 | 0.15 | 0.02 | 0.61 | 0.56 |
|  | **95% Limits of Agreement (Mean ±1.96*SD)** | **Upper** | 103.8 | 2.90 | 12.23 | 0.037 | 2.95 | 3.98 | 0.32 | 0.03 | 1.08 | 0.93 |
|  |  | **Lower** | -141.6 | -1.97 | -12.14 | -0.028 | -3.98 | -2.95 | -0.26 | -0.03 | -1.30 | -1.28 |
|  | **Linear regression (between mean and difference)** | **slope** | 0.02 | -0.16 | 0.57 | 0.103 | 1.00 | 1.00 | -0.19 | 0.03 | -0.88 | -0.20 |
|  |  | **y-intercept** | -42.82 | 10.72 | -33.41 | 0.003 | -0.82 | -0.18 | 1.52 | -0.01 | 0.11 | 0.00 |
|  |  | ***r* coefficient** | 0.01 | -0.24 | 0.45 | 0.17 | 0.61 | 0.61 | -0.29 | 0.06 | -0.63 | -0.09 |
|  |  | ***P*-value** | 0.96 | 0.27 | 0.03 | 0.45 | 0.00 | 0.00 | 0.18 | 0.78 | 0.00 | 0.68 |
|  |  |  |  |  |  |  |  |  |  |  |  |  |
| **Amygdala** | **Mean difference (between sessions)** | | -7.6 | 0.15 | -1.60 | -0.001 | -0.42 | 0.42 | 0.05 | 0.00 | -0.10 | 0.14 |
|  | **SD of difference** | | 128.8 | 1.75 | 6.51 | 0.008 | 2.51 | 2.51 | 0.16 | 0.01 | 0.58 | 0.28 |
|  | **95% Limits of Agreement (Mean ±1.96*SD)** | **Upper** | 244.9 | 3.57 | 11.15 | 0.015 | 4.50 | 5.35 | 0.37 | 0.02 | 1.04 | 0.70 |
|  |  | **Lower** | -260.0 | -3.28 | -14.35 | -0.017 | -5.34 | -4.51 | -0.28 | -0.01 | -1.23 | -0.41 |
|  | **Linear regression (between mean and difference)** | **slope** | -0.15 | -0.10 | -0.19 | -0.319 | -0.03 | -0.03 | -0.21 | 0.00 | -0.49 | -0.24 |
|  |  | **y-intercept** | 198.86 | 7.35 | 11.24 | -0.006 | 0.02 | 0.01 | 1.77 | 0.00 | 0.07 | 0.01 |
|  |  | ***r* coefficient** | -0.10 | -0.09 | -0.13 | -0.46 | -0.02 | -0.02 | -0.25 | 0.00 | -0.35 | -0.20 |
|  |  | ***P*-value** | 0.66 | 0.68 | 0.56 | 0.03 | 0.93 | 0.93 | 0.24 | 0.99 | 0.10 | 0.37 |
|  |  |  |  |  |  |  |  |  |  |  |  |  |
| **Hippocampus** | **Mean difference (between sessions)** | | -10.0 | 0.10 | -0.16 | -0.002 | -0.31 | 0.31 | -0.01 | 0.00 | -0.05 | 0.03 |
|  | **SD of difference** | | 84.5 | 1.07 | 4.23 | 0.014 | 2.10 | 2.10 | 0.14 | 0.00 | 0.46 | 0.24 |
|  | **95% Limits of Agreement (Mean ±1.96*SD)** | **Upper** | 155.5 | 2.19 | 8.13 | 0.026 | 3.81 | 4.42 | 0.28 | 0.01 | 0.84 | 0.51 |
|  |  | **Lower** | -175.6 | -2.00 | -8.45 | -0.030 | -4.42 | -3.81 | -0.29 | -0.01 | -0.95 | -0.45 |
|  | **Linear regression (between mean and difference)** | **slope** | 0.23 | 0.12 | 0.70 | 0.167 | 0.41 | 0.41 | 0.22 | 0.02 | -0.29 | -0.19 |
|  |  | **y-intercept** | -334.49 | -8.42 | -45.17 | -0.002 | -0.33 | -0.08 | -1.95 | 0.00 | 0.04 | 0.01 |
|  |  | ***r* coefficient** | 0.22 | 0.23 | 0.45 | 0.10 | 0.36 | 0.36 | 0.46 | 0.06 | -0.24 | -0.14 |
|  |  | ***P*-value** | 0.32 | 0.28 | 0.03 | 0.65 | 0.09 | 0.09 | 0.03 | 0.78 | 0.27 | 0.51 |
|  |  |  |  |  |  |  |  |  |  |  |  |  |
| **Insular cortex** | **Mean difference (between sessions)** | | -20.2 | 0.25 | -0.32 | 0.000 | -0.42 | 0.42 | 0.00 | 0.00 | -0.20 | 0.05 |
|  | **SD of difference** | | 63.1 | 0.56 | 2.26 | 0.006 | 1.56 | 1.57 | 0.10 | 0.00 | 0.57 | 0.21 |
|  | **95% Limits of Agreement (Mean ±1.96*SD)** | **Upper** | 103.4 | 1.35 | 4.11 | 0.011 | 2.65 | 3.49 | 0.20 | 0.01 | 0.93 | 0.45 |
|  |  | **Lower** | -143.9 | -0.85 | -4.74 | -0.011 | -3.48 | -2.65 | -0.20 | -0.01 | -1.33 | -0.36 |
|  | **Linear regression (between mean and difference)** | **slope** | 0.37 | -0.05 | 0.29 | -0.128 | 0.63 | 0.63 | 0.07 | 0.02 | -0.97 | -0.11 |
|  |  | **y-intercept** | -588.95 | 3.61 | -20.31 | 0.000 | -0.52 | -0.11 | -0.59 | 0.00 | 0.14 | 0.00 |
|  |  | ***r* coefficient** | 0.25 | -0.12 | 0.30 | -0.16 | 0.33 | 0.33 | 0.12 | 0.03 | -0.42 | -0.08 |
|  |  | ***P*-value** | 0.25 | 0.58 | 0.16 | 0.46 | 0.13 | 0.13 | 0.58 | 0.88 | 0.05 | 0.70 |
|  |  |  |  |  |  |  |  |  |  |  |  |  |
| **All Cortex** | **Mean difference (between sessions)** | | -7.4 | 0.33 | -0.11 | 0.000 | -0.17 | 0.17 | 0.02 | 0.00 | -0.11 | 0.09 |
|  | **SD of difference** | | 30.0 | 0.81 | 1.33 | 0.001 | 1.01 | 1.01 | 0.10 | 0.01 | 0.50 | 0.33 |
|  | **95% Limits of Agreement (Mean ±1.96*SD)** | **Upper** | 51.5 | 1.92 | 2.50 | 0.003 | 1.80 | 2.14 | 0.22 | 0.01 | 0.86 | 0.74 |
|  |  | **Lower** | -66.3 | -1.25 | -2.71 | -0.003 | -2.15 | -1.80 | -0.18 | -0.01 | -1.09 | -0.56 |
|  | **Linear regression (between mean and difference)** | **slope** | 0.06 | -0.03 | 0.27 | -0.213 | -0.05 | -0.05 | 0.07 | 0.36 | -0.96 | 1.19 |
|  |  | **y-intercept** | -92.24 | 2.34 | -15.54 | 0.001 | 0.04 | 0.01 | -0.54 | -0.06 | 0.14 | -0.04 |
|  |  | ***r* coefficient** | 0.07 | -0.06 | 0.50 | -0.34 | -0.05 | -0.05 | 0.13 | 0.47 | -0.54 | 0.76 |
|  |  | ***P*-value** | 0.76 | 0.77 | 0.01 | 0.12 | 0.81 | 0.82 | 0.56 | 0.02 | 0.01 | 0.00 |
